# Mechanistic Design of Graphdiyne‐Based Multimodal Sensing Integrating Machine Learning and Photothermal Dynamics for Precision Recognition and On‐Demand Inactivation

**DOI:** 10.1002/advs.75186

**Published:** 2026-04-09

**Authors:** Jing Xu, Hanxiao Chen, Liucun Yin, Yifang Tao, Qichen Yuan, Hong Wang, Huan Pang, Junyan Teng, Li Xue

**Affiliations:** ^1^ Department of Urology The Second Affiliated Hospital of Xi'an Jiaotong University Xi'an China; ^2^ Henan Integrative Medicine Hospital Zhengzhou China; ^3^ College of Chemistry and Chemical Engineering Xinyang Normal University Xinyang China; ^4^ School of Chemistry and Chemical Engineering Yangzhou University Yangzhou China

**Keywords:** graphdiyne, multimodal cascade, photothermal inactivation, self‐powered

## Abstract

Rapid and accurate detection of pathogenic bacteria remains essential for infection control and timely treatment. Here, we report a graphdiyne (GDY)‐based self‐powered biosensing platform that integrates CRISPR/Cas12a molecular recognition with GDY/Au nanoparticle‐engineered bioelectrodes for multimodal detection and photothermal inactivation of Vibrio parahaemolyticus. The ultrathin GDY framework provides a high‐surface‐area scaffold for uniform Au nanoparticle dispersion, facilitating interfacial charge transfer and enhancing enzyme‐mediated redox kinetics at the bioanode. Target‐triggered CRISPR/Cas12a trans‐cleavage regulates the release of glucose oxidase from a hairpin probe, enabling a self‐powered electrochemical readout driven by glucose oxidation. In parallel, HRP‐catalyzed oxidation of TMB generates oxTMB with strong near‐infrared absorbance, providing complementary colorimetric and photothermal (808 nm) outputs and enabling in situ bacterial inactivation. The electrochemical, colorimetric, and thermal modes exhibit concentration‐dependent responses with limits of detection of 0.34, 0.41, and 0.78 CFU mL^−1^, respectively. Integration of multimodal signals via machine learning further enables infection grading with an overall accuracy of 97.71%. This multimodal diagnostic‐therapeutic strategy demonstrates reliable performance in both in vitro and in vivo wound infection models, highlighting its potential for localized infection monitoring and point‐of‐care bacterial management. This study provides a proof‐of‐concept demonstration of an integrated self‐powered multimodal biosensing platform for simultaneous bacterial detection and inactivation.

## Graphical abstract

A triple‐mode theranostic platform enables integrated electrochemical, colorimetric, and photothermal detection of bacterial infection. Glucose oxidation triggers H_2_O_2_ generation and TMB oxidation to oxTMB, producing color and thermal signals under 808 nm NIR irradiation. The generated photothermal effect simultaneously eradicates bacteria, offering a sensitive detection strategy and an effective antibacterial treatment approach.

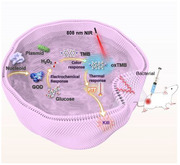

## 1 Introduction

Bacterial infections in marine environments pose an increasingly serious threat to public health, especially those caused by Vibrio parahaemolyticus (VP). After infection, symptoms such as diarrhea, vomiting, abdominal pain, and fever may occur, and in severe cases, sepsis may develop, which is particularly dangerous for individuals with weakened immune systems. Given the rapid progression of such infections, early detection and timely intervention are essential for effective disease prevention and control [1, 2]. However, although traditional detection methods have high accuracy, they are usually time‐consuming and require complex operations, which limits their application in scenarios that demand rapid response. The emergence of self‐powered biosensing platforms has completely changed the paradigm of pathogen detection, providing great advantages in sensitive detection and rapid signal response [3, 4, 5, 6]. In particular, biofuel cell‐based systems have attracted increasing attention as a promising strategy for self‐powered biosensing, as they can directly convert biochemical energy from substrates such as glucose into electrical signals for sensing and signal amplification [7, 8]. These platforms integrate biorecognition elements with advanced chemical transducers to achieve specific and portable sensitive detection of target analytes [9, 10, 11]. Therefore, self‐powered biosensors provide a sustainable and efficient solution for on‐site pathogen detection, making them highly suitable for environmental monitoring and point‐of‐care testing.

However, most existing self‐powered biosensor platforms are limited to single electrochemical detection or dual‐mode detection combining electrochemical and colorimetric methods. Although these detection modes have shown certain potential, they may still be insufficient to comprehensively identify and quantify pathogens, especially in complex samples [12, 13]. To overcome these limitations and achieve more reliable and versatile detection, there is a growing need to develop multimodal biosensors that integrate multiple detection techniques into a single platform, which can combine different detection techniques to enhance sensitivity, specificity, and versatility. The integration of thermal‐mode detection and in situ inactivation is of great significance in analytical diagnostics, particularly in the fields of pathogenic bacterial infection control and microbial monitoring [14, 15, 16, 17, 18]. Specific heat mode detection typically entails the utilization of the photothermal characteristics of the substance in question. This entails that the substance in question generates heat following its absorption of a specific wavelength of light, thereby facilitating the detection and inactivation of the target substance. In specific heat mode detection, the photothermal effect of a substance can be employed to ascertain the presence and quantity of microorganisms [19, 20]. The advantage of in situ inactivation technology is that it allows for the immediate elimination of pathogens upon detection, thereby reducing the risk of their dissemination [21, 22]. This technology is particularly well‐suited to the food processing and medical sectors, where it can rapidly respond to potential sources of infection and safeguard public health. Incorporating in situ photothermal inactivation capabilities within this multimodal strategy would enhance the safety of pathogen detection and provide a one‐stop solution for both detection and elimination of pathogens, making it highly desirable for applications in environmental monitoring and point‐of‐care diagnostics. However, research in this area is still limited, especially regarding the integration of in situ photothermal inactivation with multimodal detection strategies. While some studies have explored the use of photothermal materials for bacterial inactivation, the development of comprehensive platforms that combine detection and in situ inactivation for specific pathogens, such as VP, is rarely reported. Therefore, conducting research to develop such integrated multimodal biosensors with in situ photothermal inactivation capabilities is highly valuable and urgently needed.

VP typically exists at low concentrations, particularly during the early stages of contamination or infection. Therefore, a key challenge in the development of advanced biosensors lies in achieving high sensitivity for the detection of low levels of VP. However, current sensing platforms often fall short of the required sensitivity, especially when integrating multiple functions, such as in situ inactivation. To address this issue, the design and optimization of the sensor chip itself are of critical importance. One promising approach is to utilize nanostructured materials as the sensing substrate [23, 24, 25]. Nanostructured materials, especially 2D materials, offer unique advantages due to their high surface‐area‐to‐volume ratio, tunable physicochemical properties, and enhanced signal transduction capabilities. Graphdiyne is a novel carbon allotrope comprising alkyne linkages (sp ‐ hybridized carbon atoms) and benzene rings (sp^2^ ‐ hybridized carbon atoms). The combination of a linear sp hybrid carbon atom and a planar sp^2^ hybrid carbon atom results in the uniform distribution of pores, a π‐conjugated structure and an easily modified surface in GDY. These characteristics enhance the ability of GDY to adsorb analytes and improve the sensitivity of sensors, thereby increasing the potential for GDY applications in the field of analysis and detection [26, 27, 28]. Furthermore, the electrical conductivity and specific surface area of GDY are also pivotal factors influencing the sensing performance. The majority of reported thicknesses for GDY in analytical detection are in the range of tens of nanometres, which affects the electron transport efficiency. Furthermore, only the surface of GDY can strongly interact with the analyte. Therefore, the preparation of a thin layer of GDY with a large specific surface area is crucial to provide rich adsorption sites and obtain high electron transport efficiency, which ultimately enhances the sensor sensitivity.

In this work, we present an innovative portable self‐powered biosensor system that integrates electrical, colorimetric, and thermal detection modes, allowing for multi‐scene adaptability and rapid response. The system is based on a self‐powered sensing mechanism using GDY, a novel carbon material with unique electronic properties, in conjunction with CRISPR‐Cas12a gene editing technology [29, 30, 31, 32]. The combination enables the transformation of chemical energy into electric energy, which can power the system and facilitate electrochemical detection of VP. The system's colorimetric mode is initiated through the incorporation of the chromogenic agent TMB (3,3',5,5'‐Tetramethylbenzidine) and horseradish peroxidase, which results in the formation of oxTMB, a compound with photothermal properties. The colorimetric reaction is both portable and sensitive, enhancing the system's detection capabilities. Furthermore, the system can be activated to thermal mode by exposure to NIR radiation [33], which leads to a rapid increase in temperature, enabling in situ inactivation of VP while triggering thermal detection. A key feature of this biosensor system is its compatibility with smartphone technology, allowing for the presentation of multimodal cascade detection signals in a user‐friendly format. This accessibility is further enhanced by the integration of machine learning algorithms, which can analyze large datasets to extract features, fuse information, and classify samples with high accuracy.

## 2 Results and Discussion

### 2.1 Sensor Design and Work Mechanism

**FIGURE 1:**
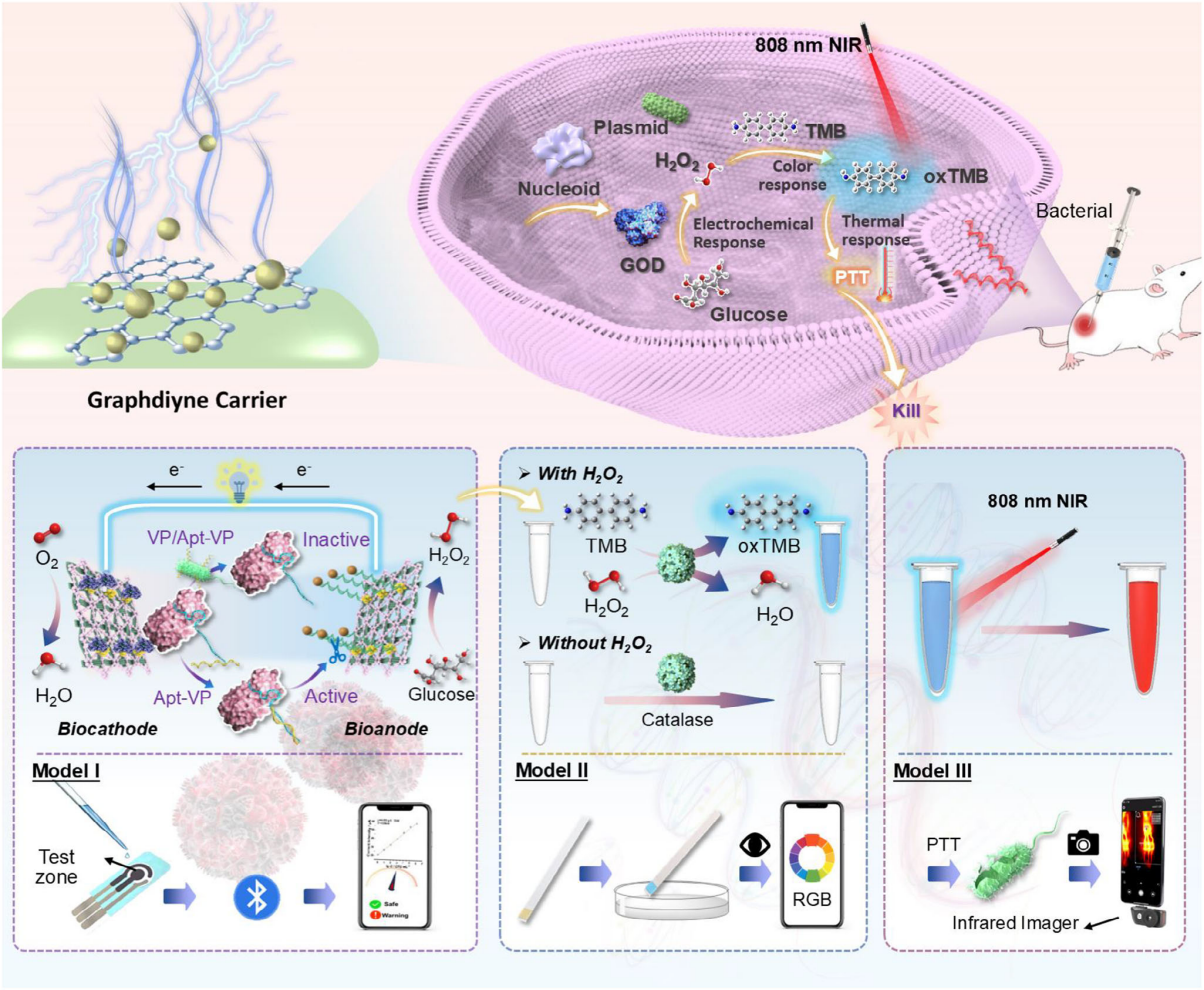
Schematic of triple‐mode self‐powered detection and in situ inactivation mechanism.

As illustrated in Figure 1, in the absence of VP, the VP‐specific aptamer (Apt‐VP) hybridizes with the guide RNA (crRNA) through base pairing, forming a crRNA/Cas12a/Apt‐VP ternary complex. This assembly activates the trans‐cleavage activity of Cas12a, enabling it to non‐specifically cleave single‐stranded DNA. When the hairpin probe‐glucose oxidase (HP‐GOD) complex is immobilized on the surface of gold nanoparticles (AuNPs), the trans‐cleavage activity of the CRISPR/Cas12a system leads to the release of GOD. In the presence of VP, Apt‐VP preferentially binds to the target bacterium, preventing the activation of Cas12a. As a result, less GOD is released, enhancing the electrochemical response at the electrode surface. This triggers the oxidation reaction of glucose at the bioanode, generating hydrogen peroxide. The resulting electrons are transported through an external circuit to the biocathode, where oxygen undergoes a reduction reaction to form water. This system converts chemical energy into electrical energy, achieving a self‐powered design. As the target concentration changes, the amount of released GOD varies accordingly, leading to corresponding alterations in the transient current value of the system. This enables electrochemical mode detection through the self‐powered system, establishing a concentration‐dependent electrical signal response mechanism.

In the colorimetric detection mode, horseradish peroxidase (HRP) catalyzes the oxidation of the chromogenic substrate TMB by hydrogen peroxide (H_2_O_2_), generating oxTMB with photothermal properties. When VP are present, the CRISPR/Cas12a system remains inactive due to the absence of target‐induced activation, preventing the release of GOD. Consequently, the HRP‐catalyzed accumulation of oxTMB triggers a series of colorimetric reactions, resulting in a visible color change in the solution. The self‐powered colorimetric detection of VP is achieved by quantitatively measuring this color shift using a colorimetric spectrophotometer. When 808 nm NIR light is introduced, the photothermal conversion capability of oxTMB enables rapid absorption of NIR light and its conversion into thermal energy, causing an instantaneous temperature rise in the system. This photothermal effect further enhances the sensitivity of the colorimetric detection, enabling three‐mode (electrochemical‐colorimetric‐photothermal) signal readout in the self‐powered biosensing platform. The entire detection strategy achieves highly sensitive detection of VP through the synergistic interaction of three modes that can be wirelessly transmitted to and analyzed via mobile devices, ensuring portability and real‐time monitoring capabilities.

The principle of high‐temperature in situ inactivation of VP relies on the bacterium's thermal sensitivity. Upon irradiation with 808 nm NIR light, the oxTMB in the system efficiently absorbs NIR light and converts it into thermal energy, inducing a rapid localized temperature increase. This rapid temperature rise effectively disrupts the cellular structure, causing protein denaturation and cell membrane rupture, thereby achieving in situ bacterial inactivation. Importantly, the oxTMB‐based system integrates colorimetric‐photothermal sensing and bacterial inactivation within a single cascade reaction, enabling simultaneous detection and treatment without the need for additional photothermal agents. This photothermal conversion‐based inactivation strategy enables simultaneous detection and direct in situ inactivation of VP within the detection system, offering an innovative solution for food safety monitoring.

### 2.2 GDY Characterization

**FIGURE 2:**
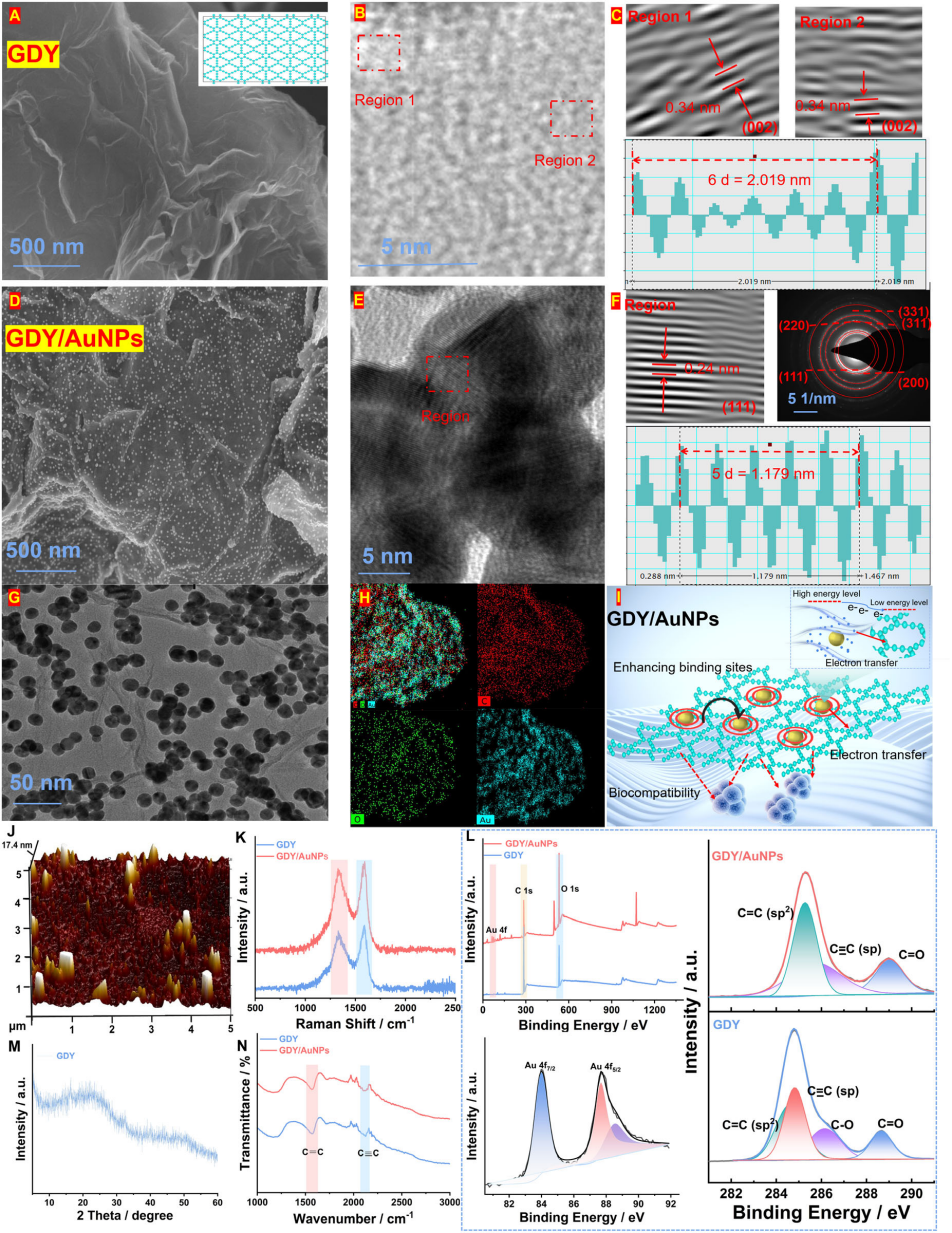
(A) SEM image of GDY, and the inset shows the structural model of GDY, (B, C) High‐magnification SEM images of GDY, (D) SEM image of GDY/AuNPs, and (E, F) high‐magnification and diffraction images of GDY/AuNPs, (G) TEM image and (H) EDS elemental mapping of GDY/AuNPs, (I) Schematic illustration of the overall mechanism of GDY/AuNPs, (J) AFM image of GDY, (K) Raman spectra of GDY and GDY/AuNPs, (L) XPS spectra of GDY and GDY/AuNPs, (M) XRD pattern of GDY, (N) FT‐IR spectra of GDY and GDY/AuNPs.

Figure 2A and Figure S1A illustrate the SEM images of the prepared thin‐layer GDY, which exhibits a typical layered curly structure. The GDY is comprised of discrete layers, each of which is clearly discernible, thereby demonstrating the material layered characteristics. Figure 2B,C shows high‐resolution spectra where the lattice spacings of 0.34 and 0.39 nm correspond to the crystalline plane structures of GDY, respectively. Figure 2D and Figure S1B present SEM images of the GDY/AuNPs composite. The bare carbon cloth (Figure S1C), with its interwoven microfiber network, offers a 3D scaffold that provides numerous attachment sites for the composite. The distribution of AuNPs on the GDY surface is very uniform, and there is no obvious aggregation or non‐uniform distribution area. The observation field reveals a dense distribution of Au NPs, which indicates that the composite has a high loading capacity. This is in stark contrast to the unsupported Au NPs prepared under identical conditions (Figure S1D), which exhibit severe agglomeration. This comparison highlights GDY's crucial role as a superior support for dispersing metal nanoparticles. Figure 2E,F shows high‐resolution images and diffraction patterns. The clear lattice fringes with a spacing of 0.24 nm correspond to the (111) planes of the face‐centered cubic gold crystal, confirming the successful formation and crystalline structure of the Au NPs. TEM image (Figure 2G) demonstrates that Au NPs loaded on GDY exhibit a diameter of approximately 10 nm, with a high loading and uniform distribution. The results corroborate the substantial specific surface area of the thin layer of GDY material and the considerable number of anchor sites. It is of particular significance that GDY/Au NPs preserves the thin lamellar structure, which markedly enhances the specific surface area of the composite. Figure 2H illustrates the TEM‐EDS of the prepared composites, indicating that the GDY/Au NPs are predominantly composed of C and Au, and are distributed in a uniform manner. Figure 2I schematically summarizes the key mechanisms, including the enhanced Au NP dispersion, efficient electron transfer, and inherent biocompatibility, which contribute to the superior performance of the GDY/Au composite. As shown in Figure 2J and Figure S2, the thickness of GDY was characterized using Atomic Force Microscopy (AFM). The GDY sample exhibited a relatively uniform thickness distribution of approximately 10.6 nm, indicating that GDY possesses an ultrathin layered structure. This structure can effectively shorten ion diffusion pathways and enhance the electron transfer rate from enzyme active sites to the electrode. Figure 2K shows the Raman spectra of GDY and the GDY/AuNPs composite, where the peak at 1382.2 cm^−1^ corresponds to the D band of GDY, arising from the breathing vibration of the sp^2^‐hybridized carbon atoms in the benzene rings. This band is associated with the structural defects of GDY. The absorption peak at 1601 cm^−1^ is attributed to the in‐plane stretching vibration of the benzene ring sp^2^ hybrid carbon atom, which is analogous to the G‐band of graphitic acetylene and is indicative of an ordered band.

XPS analysis was further employed to investigate the elemental composition and the bonding states of Au and C atoms in the GDY and GDY/AuNPs composites. Figure 2L presents the full XPS spectrum, indicating that the GDY sample primarily consists of carbon (C) and oxygen (O) elements. For comparison, the GDY/AuNPs sample shows additional Au 4f signals, confirming the successful anchoring of Au nanoparticles on the GDY surface. The high‐resolution C1s spectrum of GDY/AuNPs can be deconvoluted into three characteristic peaks corresponding to C═C, C≡C, and C═O bonds. Compared with pristine GDY, the relative intensity of the C═O component slightly increases after AuNPs loading, implying a mild surface oxidation and possible interaction between Au and oxygen‐containing groups. Furthermore, the Au 4f spectrum can be deconvoluted into two peaks located at 83.9 and 87.6 eV, attributed to Au 4f_7/2_ and Au 4f_5/2_ of metallic Au^0^, respectively, indicating that Au nanoparticles are well preserved in their metallic state after immobilization on GDY. The XRD pattern of GDY in Figure 2M shows a broad diffraction feature, indicating its low crystallinity. Figure 2N shows the FT‐IR spectrum of the GDY material, with four major absorption peaks observed at 1165, 1565, 2013, and 2120 cm^−1^, respectively. The FT‐IR spectrum of GDY/AuNPs shows almost no change compared with that of pristine GDY, indicating that the introduction of Au nanoparticles does not alter the intrinsic chemical structure of GDY. As shown in Figure S3 and the inset of Figure 2A, GDY displays a porous structure originating from its atomic arrangement, which imparts a high surface area, good chemical stability, and broad application potential.

### 2.3 Sensor Construction and Sensitive Detection

**FIGURE 3:**
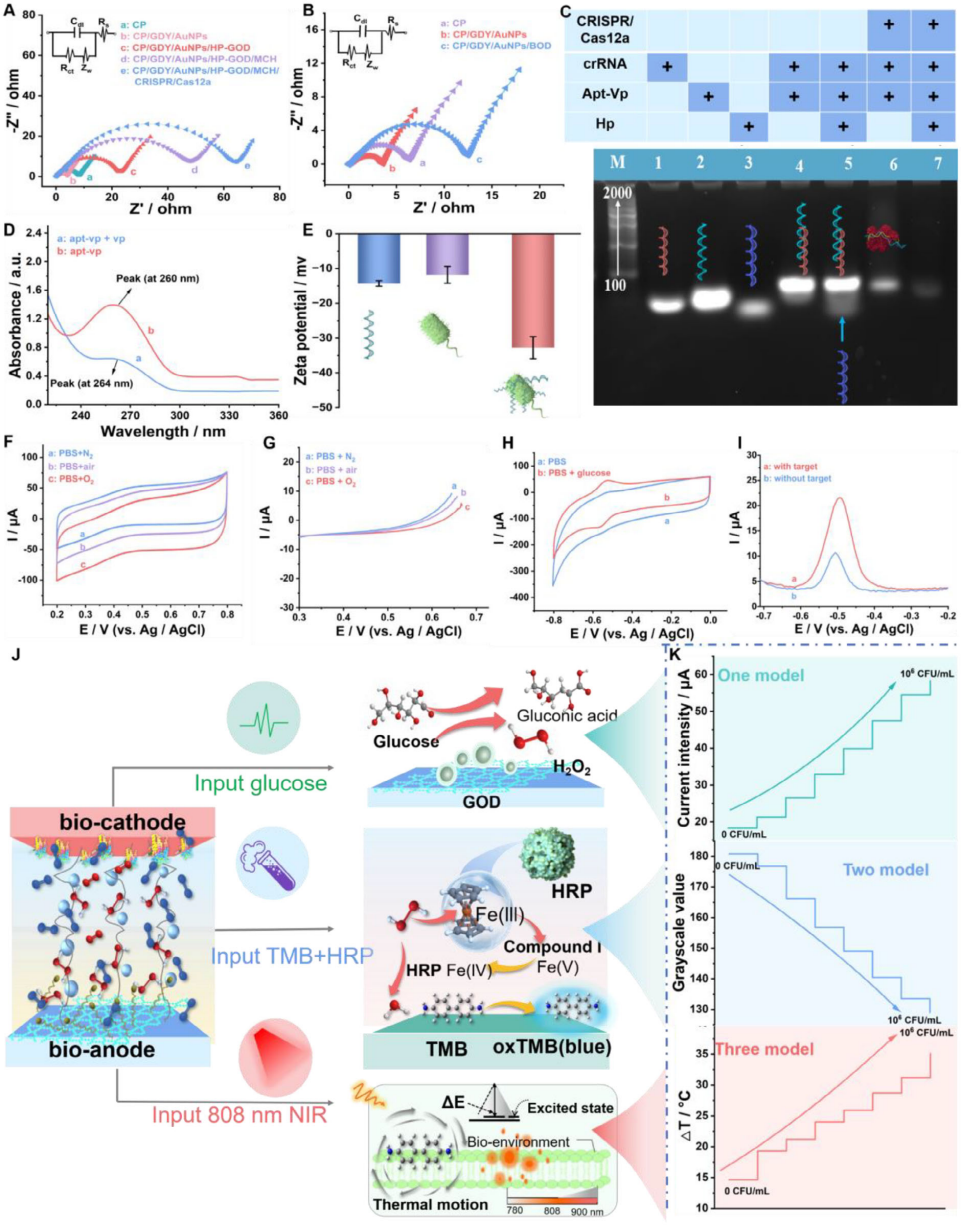
(A, B) EIS spectra of bio‐electrodes, (C) AGE for CRISPR/Cas12a reaction verify, (D, E) Validation of the binding capability between bacteria and aptamers using UV–vis spectrophotometry and laser particle size analysis, (F) cyclic voltammetry (CV) curve of the bio‐cathode, (G) Linear sweep voltammetry (LSV) response of the biocathode in different electrolyte solutions, (H) CV response of the bioanode in different electrolyte solutions, (I) differential pulse voltammetry (DPV) curve of the bio‐anode, (J) Schematic illustration of the sensing mechanism based on three synergistic signal transduction modes, (K) Concentration‐dependent signal responses of the integrated electrochemical, colorimetric, and photothermal detection modes.

The DNA and RNA sequences used for sensor construction are listed in Table S1. Figure 3A,B shows the changes in impedance values of the bioanode and cathode. All curves are composed of a semicircular arc and an oblique line, indicating that the modification of the bio‐electrode has a similar reaction mechanism. The diameter of the semicircle represents the charge transfer resistance (Rct). Compared with the carbon paper (CP) substrate (curve a), the semi‐arc is significantly reduced after the GDY/AuNPs modification (curve b), indicating that the GDY/AuNPs composite has excellent electrical conductivity and is conducive to electrochemical reaction. After the modification of the bioanode with MCH and the biocathode with bilirubin oxidase (BOD) (curve c), a significant increase in Rct is observed. This can be attributed to the nonspecific electrostatic repulsion caused by MCH and the pronounced steric hindrance effect of the protein, both of which impede the electron transfer process. Furthermore, the impedance of the bioanode gradually increased following the stepwise modification of the biofunctional strategy (curves d‐e), due to the electronegativity of the phosphate backbone and the repulsion of electrons. The feasibility of the CRISPR/Cas12a reaction is verified by Agarose Gel Electrophoresis (AGE). As shown in Figure 3C, three distinct bands are observed in channels 1–3, representing crRNA, Apt‐VP, and HP. The strip of channel 4 represents the double strand formed as a consequence of the hybridization of crRNA and Apt‐VP, while the two strips of channel 5 represent target HP and the aforementioned double strand, respectively. The formation of the crRNA/Cas12a/Apt‐VP ternary complex was demonstrated in channel 6, while the addition of the HP probe did not result in a change to the number of bands observed in channel 7. The results indicate the effective of CRISPR/Cas12a trans‐cutting activity. The binding capacity between bacteria and aptamers was validated using UV–vis spectrophotometry and laser particle size analysis. In its free state, Apt‐VP exhibits a loose single‐stranded structure with numerous exposed bases. Upon binding to bacteria, the Aptamer undergoes folding to form a compact 3D structure. This structural transition enhances base stacking interactions and reduces the probability of electronic transitions, consequently leading to a decrease in absorbance at 260 nm (Figure 3D). Apt‐VP carries a negative charge due to its phosphate backbone. VP also predominantly displays a negatively charged surface owing to the presence of lipopolysaccharides in the outer membrane and negatively charged functional groups. When the aptamer binds to the bacterial surface, it may alter the overall charge distribution by forming a negative charge shield through Aptamer–bacteria interactions, thereby resulting in a reduction of Zeta potential (Figure 3E). As illustrated in Figure 3F, the reduction peak potential demonstrates an increase with elevated oxygen content, thereby substantiating the oxygen‐mediated reduction reaction. Figure 3G presents the LSV curves of the biocathode in various testing solutions, which are consistent with the CV results. Figure 3H and Figure S4 show the CV and LSV curves of the bioanode in PBS solution with and without glucose. It is clearly observed that the typical oxidation peak of curve b is stronger than curve a, indicating the catalytic activity of GOD. Figure 3I depicts the DPV curves of the bioanode with (curve a) and without (curve b) the target. The anode peak current value exhibits a pronounced surge with the introduction of the VP, thereby confirming the sensitivity of the bioanode to the target.

As shown in Figure 3J, the sensing platform operates through three synergistic signal transduction modes. In the first (electrochemical) mode, glucose is catalytically oxidized by GOD at the bio‐anode to produce gluconic acid and H_2_O_2_. In the second (colorimetric/electrocatalytic) mode, HRP utilizes the generated H_2_O_2_ to oxidize TMB into oxTMB, accompanied by a Fe(III)→Fe(IV) = O→Fe(V) = O redox cycle and a distinct color change. In the third (photothermal) mode, the produced oxTMB exhibits strong near‐infrared absorption; under 808 nm irradiation, molecular excitation and nonradiative relaxation generate a measurable photothermal effect. The synergistic operation of these three modes enables multi‐signal outputs‐current, colorimetric, and thermal responses‐thereby significantly enhancing the sensitivity and reliability of detection. To further evaluate the self‐powered capability of the platform, the open‐circuit voltage (E^OCV^) of the biofuel cell was measured under standard conditions without any external load. As shown in Figure S5, the voltage remained stable throughout the measurement, indicating that the system can generate electrical energy autonomously, without the need for an external power supply. As shown in Figure 3K, the signal values exhibit a clear concentration‐dependent relationship in all three detection modes. With the increase of VP concentration, the reduced formation of the ternary complex suppresses the CRISPR/Cas12a cleavage of HP‐GOD on the bioanode, thereby decreasing GOD release and enhancing the electrical signal detected by the smartphone. The linear equation is y = 6.533 lg C + 20.50, and the detection limit (LOD, *S/N* = 3) is 0.34 CFU/mL (Figure S6). Based on the above, a colorimetric strategy was employed to measure VP at different standard concentrations. As the VP concentration increased, the solution's color gradually deepened, and the grayscale value decreased accordingly. A linear relationship was observed between VP concentration and both the grayscale value and absorbance within the concentration range of 1 to 10^6^ CFU/mL. The resulting linear regression equation is y = −7.565 lg C + 172.4, and the calculated LOD is 0.41 CFU/mL (Figure S7). The absorbance of the oxidized TMB at 652 nm increased (Figure S8A,B), indicating a corresponding enhancement in the color intensity of the TMB oxidation product. To verify that the observed temperature rise was indeed caused by oxTMB, we performed a control experiment: as shown in Figure S9, PBS and TMB alone exhibited negligible temperature increases under NIR irradiation, whereas oxTMB produced a significant photothermal effect, indicating that the thermal signal originates from oxTMB. Moreover, a pronounced temperature rise was observed only in the presence of bacteria, confirming that the photothermal response is associated with bacteria‐triggered oxTMB formation. This is attributed to the reduction in GOD released from the electrode with increasing VP concentration, leading to an accumulation of the product (H_2_O_2_) generated from glucose oxidation. In colorimetric mode, oxTMB products with photothermal properties open a channel for thermal mode detection, and the application of NIR leads to a swift rise in temperature, enabling thermal detection. In Figure S10, the concentration of VP is found to be linearly related to temperature, with a linear equation of ΔT = 2.560 lg C + 18.75, with the LOD of 0.78 CFU/mL. The above results confirm that the experiment uses enzyme‐based biofuel, GDY nanomaterials, and CRISPR‐Cas12a technology to accurately detect VP through three models. Each mode shows a linear relationship with VP concentration, indicating that the system can detect different concentrations of VP sensitively and accurately. It should be noted that the term “self‐powered” in this work specifically refers to the electrochemical sensing mode driven by the glucose oxidation reaction without an external power supply, while the photothermal mode under NIR irradiation serves as an auxiliary readout and therapeutic function.

### 2.4 Reproducibility, Stability, Specificity

**FIGURE 4:**
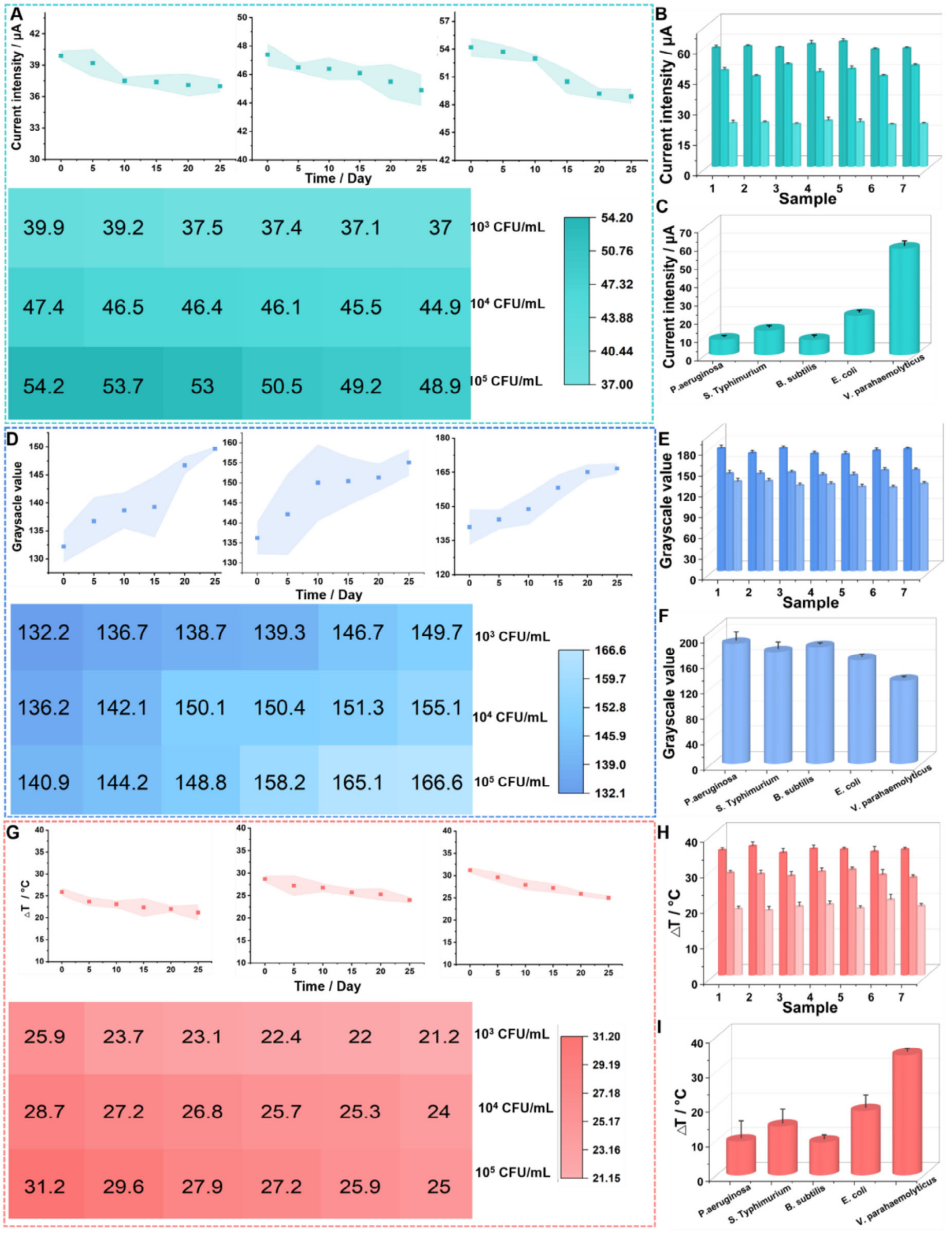
Electrochemical mode: (A) stability, (B) repeatability at a target concentration of10^2^ ,10^4^ and 10^6^ CFU mL^−1^, and (C) specificity toward VP, Colorimetric mode: (D) stability, (E) repeatability at a target concentration of 10^2^ ,10^4^ and 10^6^ CFU mL^−1^ and (F) specificity toward VP, Thermal mode: (G) stability, (H) repeatability at a target concentration of 10^2^ ,10^4^ and 10^6^ CFU mL^−1^ and (I) specificity toward VP.

To evaluate the performance of the sensors, self‐powered sensing chips that were stored at 4°C for different durations were tested. As shown in Figure 4A, the current response in the electrochemical mode remained at approximately 90% of its initial level after 25 days, confirming excellent stability. Notably, even when stored at room temperature (25°C), the sensor chips retained ∼90% of their initial signal after 7 days, demonstrating good short‐term stability without special storage requirements (Figure S11). Figure 4B shows the reproducibility of the electrochemical mode: seven independent sensors, tested at VP concentrations of 10^0^, 10^4^, and 10^6^ CFU/mL exhibited relative standard deviations (RSDs) below 5%. The specificity of the electrochemical mode is shown in Figure 4C, where significant signal changes were observed only in the presence of VP, but not with four common bacterial interferents (Pseudomonas aeruginosa, Salmonella typhimurium, Bacillus subtilis, and Escherichia coli). For the colorimetric mode, Figure 4D shows that the grayscale value remained at a high level after 25 days of storage. The reproducibility (Figure 4E) and specificity (Figure 4F) similarly confirmed good performance, with only VP producing significant signal changes. Additionally, the repeatability of the sensor in the colorimetric detection mode was evaluated using UV–vis absorption spectroscopy (Figure S12A) at a VP concentration of 10^2^ CFU mL^−1^. The relative standard deviation (RSD) between different sensors was 2.37%, consistent with the results obtained in the electrochemical mode, further confirming the high repeatability of the sensor chip. Meanwhile, the sensor exhibited good specificity toward VP in the colorimetric mode (Figure S12B). For the thermal mode, Figure 4G shows stable temperature responses after 25 days, while reproducibility (Figure 4H) and specificity (Figure 4I) confirmed consistent and target‐specific performance. Compared with previously developed VP detection methods, the self‐powered biosensor constructed in this study, based on GDY as the substrate, exhibited superior performance in terms of detection limit, linear range, and response time (Table S2).

### 2.5 Analysis of Real Samples

To assess the applicability of the constructed self‐powered biosensing platform in real‐world samples, the standard addition method was employed to determine the concentration of VP in various water samples, including river water collected from different locations (sampling temperature: 31°C). As shown in Tables S3–S5, the spiked recovery rates for the electrochemical method ranged from 94.48% to 102.1%, for the colorimetric method from 97.54% to 107.3%, and for the specific heat‐based method from 97.70% to 102.3%. Moreover, no significant differences were observed among the results obtained by the three methods, indicating the great potential of the biosensor for practical sample analysis. To evaluate the practical applicability of the proposed sensor in complex biological matrices, spike‐recovery experiments were performed in diluted human serum samples spiked with different concentrations of Vibrio parahaemolyticus. As shown in Table S6, the recoveries ranged from 96.66% to 112.4%, with RSD values below 6%, indicating satisfactory accuracy and precision. These results demonstrate that the sensor maintains reliable performance in complex serum environments and exhibits excellent anti‐interference capability for detecting Vibrio parahaemolyticus in real samples.

### 2.6 In Situ Inactivation

**FIGURE 5:**
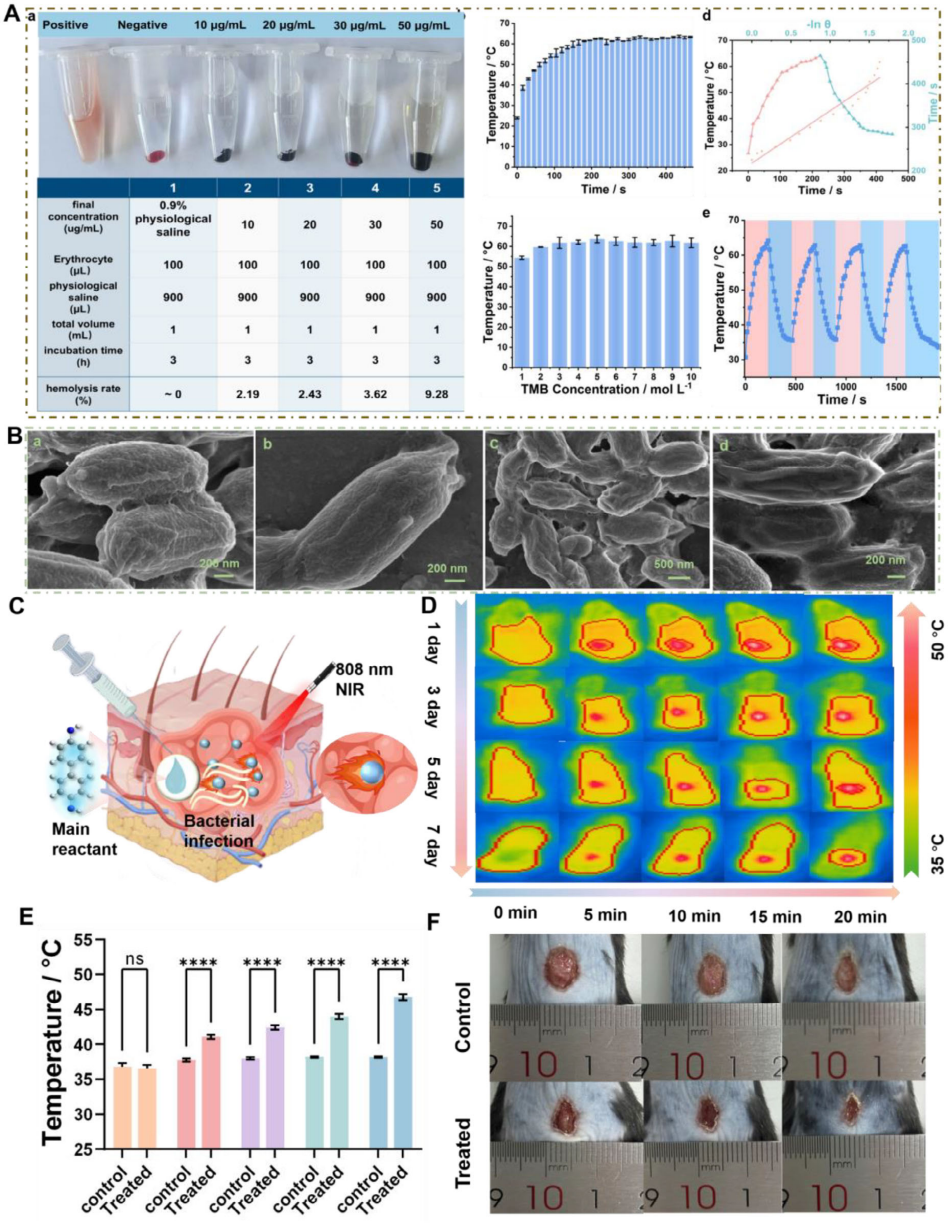
(A) Analysis of GDY hemolysis experiment, (B) Infrared thermal imaging under different irradiation durations, (C) Infrared thermal imaging under different concentrations, (D) The cyclic heating effect of the oxTMB solution, (E) Photothermal conversion capability of oxTMB aqueous solution and time constant calculation for the cooling cycle, (F, G) SEM images of V. parahaemolyticus cell morphology before and after inactivation, (H) Schematic diagram of the wound infection photothermal therapy model, (I) Infrared thermal images of experimental group mice under irradiation at different time points, (J) Differential analysis plots comparing control and experimental groups across irradiation durations, (K) Comparative visualization of wound healing progression between control and experimental groups at post‐treatment intervals.

As shown in Figure 5A, the biocompatibility of GDY was evaluated through a hemolysis assay. With increasing GDY concentrations (0–50 µg mL^−1^), the absorbance at 540 nm gradually increased (Figure S13). However, the hemolysis ratio remained within the safe range throughout the tested concentration range, indicating the good biocompatibility of GDY. Furthermore, the hemolytic behavior of the GDY/AuNPs composite was also examined. As shown in Figure S14, no obvious hemoglobin absorption signal was observed within the concentration range of 10–100 µg mL^−1^, and the calculated hemolysis ratios were below 5%, indicating that the composite system also possesses good hemocompatibility. The photothermal behavior of GDY/AuNPs under NIR irradiation was then evaluated, and the maximum temperature increased with increasing concentration (Figure S15). Considering both the photothermal performance and the compatibility with the sensing interface, 100 µg mL^−1^ was chosen as the optimal working concentration for subsequent experiments. At this concentration, the photothermal conversion efficiency of GDY/AuNPs was determined to be 40.87%, indicating efficient NIR photothermal conversion (Figure S16). In this system, GDY/AuNPs play a synergistic role, promoting thermal signal output and thereby contributing to improved antibacterial performance. As depicted in Figure 5B,C, infrared thermal imaging was employed to monitor temperature variations under different concentrations and irradiation durations. The highest temperature was achieved with 5 mM oxTMB under 225 s irradiation. The time‐temperature profiles from on/off cycling experiments (Figure 5D) revealed that the temperature peaks remained consistently above 60°C throughout four consecutive cycles, demonstrating the excellent photothermal stability of oxTMB. Furthermore, the photothermal conversion efficiency of 5 mM TMB was calculated to be 47.36% (Figure 5E). To further investigate the impact of the photothermal treatment on bacterial cells, morphological changes before and after inactivation were examined. As shown in Figure 5F,G, notable morphological discrepancies were discerned between the bacterial specimens prior to and following inactivation. Prior to inactivation (Figure 5F), the bacterial cells exhibited a relatively full and rounded form, which indicates the enhanced internal cellular structure and robust cellular activity. Following inactivation (Figure 5G), a notable alteration in the morphology of the bacteria was observed. The bacterial cells were markedly diminished in size, giving the overall cell an emaciated and shriveled appearance. This alteration may be attributed to the destruction or loss of water and nutrients within bacterial cells during the inactivation process, which ultimately leads to the collapse of the cell's internal structure and the relaxation of the cell wall [30]. This observation supports the conclusion that oxTMB has a pronounced antibacterial effect. This striking contrast serves to illustrate the impact of the inactivation treatment, and demonstrates the pronounced antibacterial efficacy of oxTMB. Furthermore, it offers an effective visual means of evaluating the efficacy of antimicrobials.

As shown in Figure 5H, the solution was injected into the wounds of experimental group mice after turning deep blue, followed by irradiation with an 808 nm laser (1.0 W/cm^2^) to validate the antibacterial performance. Figure 5I,J displays temperature variations recorded by an infrared thermal imaging system during different treatment days. Significant differences between the control group and experimental group were observed immediately after irradiation initiation, indicating that the photothermal therapeutic purpose could be achieved in the experimental group mice. During the treatment process, a comparison of wound healing between the experimental and control groups (Figure S17) in conjunction with Figure 5K, revealed that after seven days of treatment, the wound area in the experimental group was significantly reduced. This result confirms the antibacterial efficacy conferred by the photothermal effect. As shown in Figure S18A,B, a complete blood count (CBC) analysis was performed to evaluate the biosafety of this photothermal therapy in mice. Red blood cell‐related parameters, including mean corpuscular hemoglobin concentration (MCHC) and red blood cell distribution width‐coefficient of variation (RDW‐CV), were measured and compared between the control and treatment groups. All values were within the normal physiological range, and no significant differences were observed between the two groups, indicating that the therapy induced no detectable hematological toxicity or systemic adverse effects during this antibacterial assay. CBC parameters of both groups were systematically analyzed using a Mindray veterinary automatic hematology analyzer (Figure S19A,B). The results revealed that key indicators in the treatment group, including granulocyte count (Gran#), hemoglobin (HGB), and hematocrit (HCT), remained within normal physiological ranges, confirming the therapeutic efficacy of the photothermal treatment.

### 2.7 Monitoring and Prediction of Bacterial Infections Assisted by Machine Learning

**FIGURE 6:**
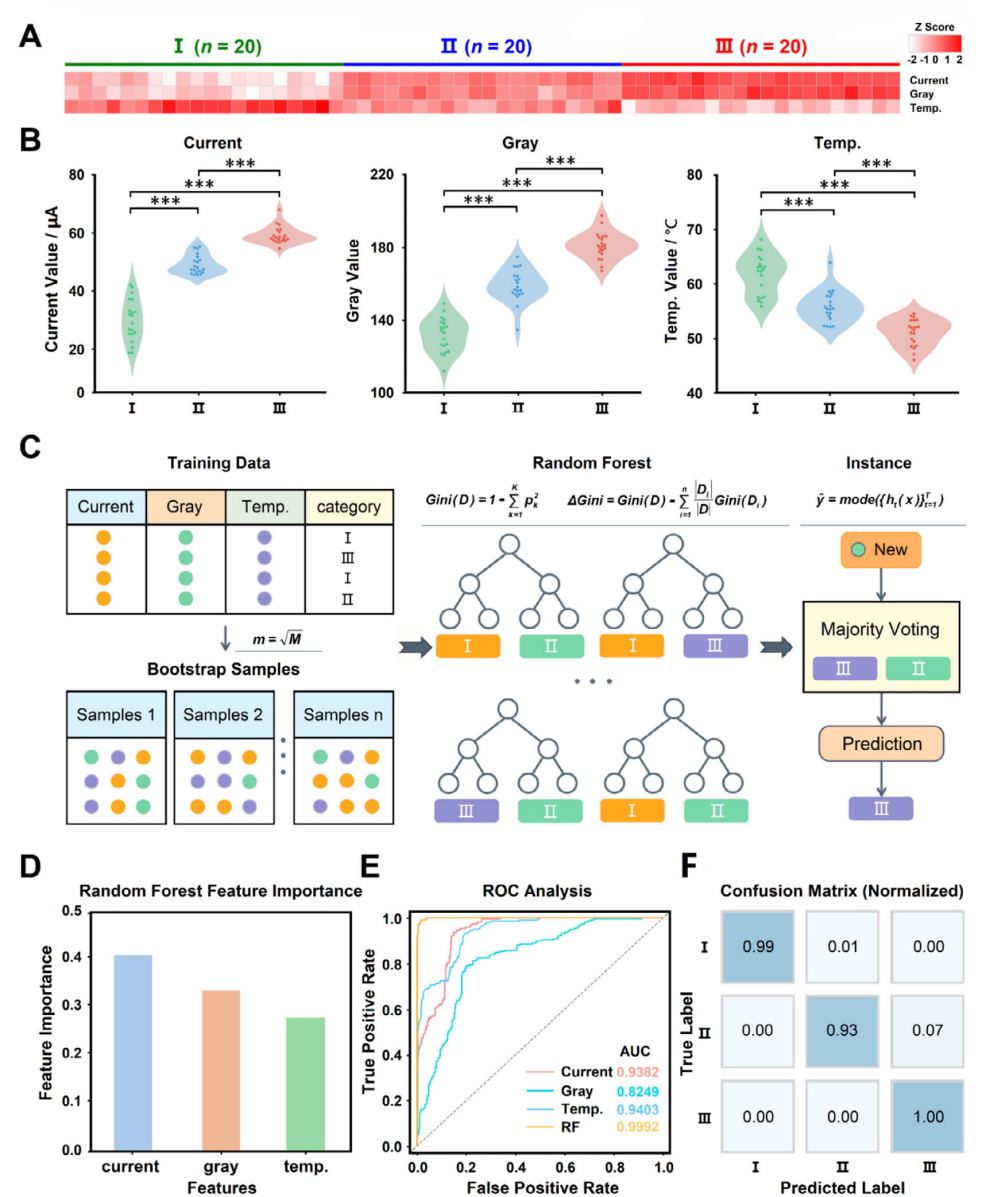
Workflow of machine learning‐based modeling and evaluation for bacterial infection grading. (A) Heatmap of the training phase showing the expression profiles of characteristic features for infection Grades I, II, and III. (B) Following the Shapiro‐Wilk normality test, differences among feature variables were analyzed using either independent‐sample t‐tests or Mann‐Whitney U tests depending on the data distribution (***p < 0.001). (C) Schematic diagram of the random forest model construction process. (D) Feature importance ranking in the random forest classification model. (E) ROC curve analysis of individual features and their discriminatory performance within the model. (F) Confusion matrix indicating that the random forest model achieved a classification accuracy of 97.71% across samples from Grades I, II, and III.

We classified the severity of infection into three levels (Level I, Level II, and Level III) and analyzed a large dataset, with an equal number of samples in each category. A total of 300 independently prepared samples (∼35 per concentration) were measured in triplicate after signal stabilization and averaged for model training. The dataset was divided into training and test sets using stratified random sampling prior to model development, ensuring balanced concentration distributions while preventing information leakage. As shown in Figure 6A,B, there were statistically significant differences in the characteristics across the three levels (***p<0.001). To effectively handle high‐dimensional data and identify nonlinear relationships, we selected the Random Forest algorithm, as shown in Figure 6C, as the primary classifier. In the training set, the accuracy reached 100%. According to Figure 6D, the current values contributed most to the model classification. Furthermore, ROC analysis (Figure 6E) and the confusion matrix (Figure 6F) demonstrated that the model exhibited excellent generalization performance on the validation set: the overall AUC value was 0.9951, significantly outperforming any single feature. The confusion matrix analysis results, as shown in Figure 6F, were as follows: for Level I, the specificity was 100% and sensitivity was 99.35%; for Level II, the specificity was 99.39% and sensitivity was 92.59%; for Level III, the specificity was 98.08% and sensitivity was 100%. The overall accuracy was 97.71%, confirming the practical application potential of this model in bacterial infection risk assessment and grading.

## 3 Conclusion

In summary, we developed a multimodal, self‐responsive biosensing platform for integrated diagnosis and treatment of pathogenic infections. By combining CRISPR/Cas12a‐mediated recognition, graphdiyne‐based biofuel‐powered energy generation, and NIR‐triggered photothermal conversion, the system enables self‐powered detection through electrochemical, colorimetric, and thermal modalities. The platform demonstrates high sensitivity, excellent specificity, and effective antibacterial performance in both in vitro and in vivo experiments. Furthermore, the integration of machine learning improves infection risk stratification, highlighting its potential for intelligent diagnostic applications. Although the current configuration is mainly suitable for surface infections or environmental samples, future efforts will focus on system miniaturization and integration with wearable and IoT‐enabled devices for continuous monitoring. This strategy provides a promising foundation for next‐generation point‐of‐care diagnostics and precision infection management.

## 4 Experimental Section

### 4.1 Synthesis of GDY

Synthesize GDY according to the method described in the Supporting Information.

### 4.2 Synthesis of Au NPs/GDY Composite

A volume of 5 mL of GDY solution was ultrasonicated for 20 min, followed by the addition of 100 µL of 1% sodium citrate and 50 µL of 1% HAuCl_4_ solution. The mixture was stirred at 70°C for 1 h, and then centrifuged at 8000 rpm. The precipitate was washed three times with ultrapure water and subsequently redispersed in DMF to obtain the Au NPs/GDY solution, which was stored at 4°C.

### 4.3 Synthesis of CRISPR/Cas12a

Cas12a (25 µL, 100 nm), guide RNA (crRNA, 25 µL, 120 nm), and 1× reaction buffer were mixed and incubated at 25°C for 30 min to assemble the CRISPR/Cas12a system.

### 4.4 Construction of Bioanode and Biocathode

Prepare the bioanode and biocathode according to the method described in the Supporting Information.

### 4.5 Fabrication of the Self‐Powered Biosensor

The testing buffer consisted of 30 mL of phosphate‐buffered saline (PBS, pH 7.4) containing 5 mmol/L glucose. Target bacteria at varying concentrations were incubated at 37°C for 30 min. Upon addition of the target bacteria, little to no release of Apt‐VP occurred, and the HP‐GOD remained immobilized on the surface of the bioanode, resulting in a high signal response; the corresponding transient current was recorded. In the absence of the target, the CRISPR/Cas‐mediated biosensing strategy was activated via trans‐cleavage, triggering the release of GOD and causing a decrease in the current signal. The transient current signal, amplified by a supercapacitor, was transmitted to a digital multimeter (DMM) connected via Bluetooth to a smartphone for real‐time monitoring.

### 4.6 Calculation of Photothermal Conversion Efficiency

The photothermal conversion efficiency of TMB (5 mm) oxidized by hydrogen peroxide generated at the bioanode was calculated according to previously reported methods, as described below:

$$\eta =\frac{h_{s}\left(T\max-Tsur\right)-Q_{d_{i}s}}{I\left(1-10^{-A}\right)}$$

$$\tau_{s}=m_{D}c_{D}/\mathrm{hs}$$

$$T=-\tau_{s}\;\ln\theta$$

$$\theta =\frac{T-Tsur}{T\max-Tsur}$$

here, the *τ*_s_ value was 120.46 (J W^−1^), *Q*_dis_ (mW) represents the heat loss due to light absorption by the container; A is the absorbance of the solution at 808 nm; h (W·cm^−2^·K^−1^) is the heat transfer coefficient; T (K) denotes the temperature as a function of time; *T*_sur_ (K) is the ambient temperature; *T*_max_ (K) is the maximum temperature of the solution; I (W) is the laser power; s (cm^2^) is the surface area of the container opening; τ (s) is the system time constant; m_D_ (g) and c_D_ (J·g^−1^·K^−1^) are the mass and specific heat capacity of the solvent, respectively.

### 4.7 Ethics Statement

All human serum samples used in this study were collected in accordance with ethical requirements and strictly followed relevant medical guidelines. All procedures for sample collection and use were approved by the Medical Ethics Committee of the Second Affiliated Hospital of Xi'an Jiaotong University (Approval No. 2025YS381). All animal experiments were approved by the biomedical ethics committee of health science center of Xi'an Jiaotong University (Approval No.: XJTUAE2025.1016) and were conducted under the authorization for biomedical research involving animals.

## Conflicts of Interest

The authors declare no conflicts of interest.

## Supporting information




**Supporting File**: advs75186‐sup‐0001‐SuppMat.docx.

## Data Availability

The data and materials are all included in this manuscript.
