# Supplementary material for: Mechanistic Design of Graphdiyne‐Based Multimodal Sensing Integrating Machine Learning and Photothermal Dynamics for Precision Recognition and On‐Demand Inactivation
Source: Adv Sci (Weinh). 2026 Apr 9:e75186. Online ahead of print. doi: 10.1002/advs.75186 (PMC13334649; doi:10.1002/advs.75186)
Supplement: Supplementary file 1 — Supporting File: advs75186‐sup‐0001‐SuppMat.docx. [file ADVS-9999-e75186-s001.docx]

*Supporting Information*

**Mechanistic Design of Graphdiyne-Based Multimodal Sensing Integrating Machine Learning and Photothermal Dynamics for Precision Recognition and On-Demand Inactivation**

Jing Xu^1^, Hanxiao Chen^1,3,4^, Liucun Yin^1^, Yifang Tao^1^, Qichen Yuan^1^, Hong Wang^1^, Huan Pang^2*^, Junyan Teng^3*^, Li Xue^1^*

^1^ Department of Urology, The Second Affiliated Hospital of Xi'an Jiaotong University, Xi’an 710004, China

^2^ School of Chemistry and Chemical Engineering, Yangzhou University, Yangzhou 225009, China
^3^ Henan Integrative Medicine Hospital 45004, Zhengzhou, China

^4^ College of Chemistry and Chemical Engineering, Xinyang Normal University, Xinyang 464000, China.

*Corresponding author to one of the following:

Huan Pang, Yangzhou University, Email: [huanpangchem@hotmail.com](mailto:huanpangchem@hotmail.com)
Junyan Teng, Henan Integrative Medicine Hospital 45004, Zhengzhou, China, Email: tengjy1981@163.com

Li Xue, The Second Affiliated Hospital of Xi'an Jiaotong University, Email:

[xueli1979@xjtu.edu.cn](mailto:xueli1979@xjtu.edu.cn)

**Chemicals**

The DNA sequences of oligonucleotides (Table S1) were purchased from Shanghai Shenggong Biotechnology Co., Ltd. Pseudomonas aeruginosa (ATCC 27853), Salmonella enterica serovar Typhimurium (ATCC 14028), Vibrio parahaemolyticus (ATCC 17802), Bacillus subtilis (ATCC 6633), and Escherichia coli (ATCC 25922) were all purchased from Beijing Luqiao Technology Co., Ltd. 1-ethyl-3-(3’-dimethylaminopropyl) carbodiimide (EDC, ≥ 98%), N hydroxysuccinimide (NHS, ≥ 98%), glucose oxidase (GOD, BR, 100-250 U/mg), diethylprocarbonate (DEPC, ≥ 99%), chloroauric acid tetrahydrate (HAuCl_4_·3H_2_O, ≥ 99%), 4-(N maleimidomethyl) cyclohexane-1-carboxylic acid 3-sulfo-N-hydroxysuccinimide ester sodium salt (sulfo-SMCC, ≥ 99%), phosphate buffered saline (PBS), sodium citrate (≥ 99%), monosodium dihydrogen phosphate (NaH_2_PO_4_, ≥ 99%), ethanol (C_2_H_6_OH, ≥ 99%), and disodium hydrogen phosphate (Na_2_HPO_4_, ≥ 99%) were obtained from Sinopharm Chemical Reagent Co. Ltd (Shanghai, China). The carbon paper (CP) (WOS1009) was purchased from Sigma-Aldrich. Potassium ferrocyanide trihydrate (K_4_[Fe(CN)_6_], ≥ 99.95%), potassium ferricyanide (K_3_[Fe(CN)_6_], ≥ 99.5%), potassium chloride (KCl, ≥ 99. 5%), NaOH (≥ 97%), 6-Mercapto-1-hexanol (MCH, 97%), acetic acid (CH_3_CH_2_COOH, ≥ 99.7%), sodium acetate solution (CH_3_CH_2_COONa, 99%), 3,3',5,5'-Tetramethylbenzidine (TMB, 99.0%), sodium chloride (AR, 99.5%), tris buffer solution (0.01 M) were purchased from McLean Biochemical Reagent Co., Ltd. Horseradish peroxidase (HRP, ≥ 250 U/mg) was obtained from Aladdin Biochemical Technology Co., Ltd.

The [Fe(CN)_6_]^3-/4-^ solution for the electrochemical tests consisted of 10 mM K_4_Fe(CN)_6_, 10 mM K_3_Fe(CN)_6_, and 0.1 M KCl. The buffer solutions for the experiment were as follows: phosphate buffered solutions (PBS) consisted of 0.1 M NaCl, 0.1 M Na_2_HPO_4_, and 0.1 M NaH_2_PO_4_ (pH 7.4).

**Experimental Section**

Two-dimensional (2D) ultrathin GDY/G nanostructures were fabricated by employing liquid-phase exfoliated graphene (G) as an epitaxial growth template in a solution containing hexakisethynylbenzene. GDY was epitaxially grown on both sides of the G sheets. The resulting GDY/G exhibited lower electrochemical impedance and a higher electrochemically active surface area, both of which are beneficial for mass diffusion and electron transfer. When employed as an electrode material in electrochemical sensors, GDY/G demonstrates enhanced performance due to d-π and π-π interactions (*1*).

**Synthesis of Au NPs**

Gold nanoparticles (Au NPs) were synthesized using the sodium citrate reduction method [2]. Briefly, 100 mL of 10% HAuCl_4_·3H_2_O solution was diluted with 50 mL of ultrapure water and heated to boiling. Then, 100 μL of 1% sodium citrate solution was added to the boiling mixture under continuous stirring. The solution was stirred until it turned wine-red, and heating was maintained for an additional 10 minutes. After cooling to room temperature, the resulting Au NPs suspension was stored at 4 °C in the dark for further use.

**Construction of Bioanode and Biocathode**

To prepare the bioanode

50 μL of 1 mg/mL Au NPs/GDY solution was dropped onto the surface of a carbon paper (CP) electrode (1 × 1 cm^2^) and vacuum-dried at 37 °C for 2 hours. The modified electrode was then immersed in 40 μL of a 1 mg/mL EDC/NHS mixture and incubated at room temperature for 40 minutes. After washing off the excess EDC/NHS with ultrapure water, 50 μL of 1 mmol/L HP probe was added and incubated at 4 °C for 10 hours. Subsequently, 40 μL of 1 mmol/L MCH was added and incubated for 30 min to obtain the MCH/HP-GOD/GDY/Au NPs/CP bioanode.

To prepare the biocathode

50 μL of 1 mg/mL AuNPs/GDY solution was dropped onto the surface of a CP electrode (1 × 1 cm^2^) and vacuum-dried at 37 °C for 2 hours. The electrode was then immersed in 80 μL of 1 mg/mL EDC/NHS mixture and incubated at room temperature for 30 minutes. After rinsing with ultrapure water to remove excess EDC/NHS, 50 μL of 5 mg/mL BOD solution was dropped onto the activated electrode surface and incubated at 4 °C for 10 hours. The electrode was then washed with ultrapure water and stored at 4 °C, resulting in the BOD/AuNPs/GDY/CP biocathode.

**Statistical Analysis**

Certain datasets were analyzed using GraphPad Prism 9.0 software, with results expressed as mean ± standard deviation (SD). One-way analysis of variance (ANOVA) was employed to assess statistical significance among groups (*P < 0.05, **P < 0.01, ***P < 0.001, ****P < 0.0001). A value of P < 0.05 was considered statistically significant.

**
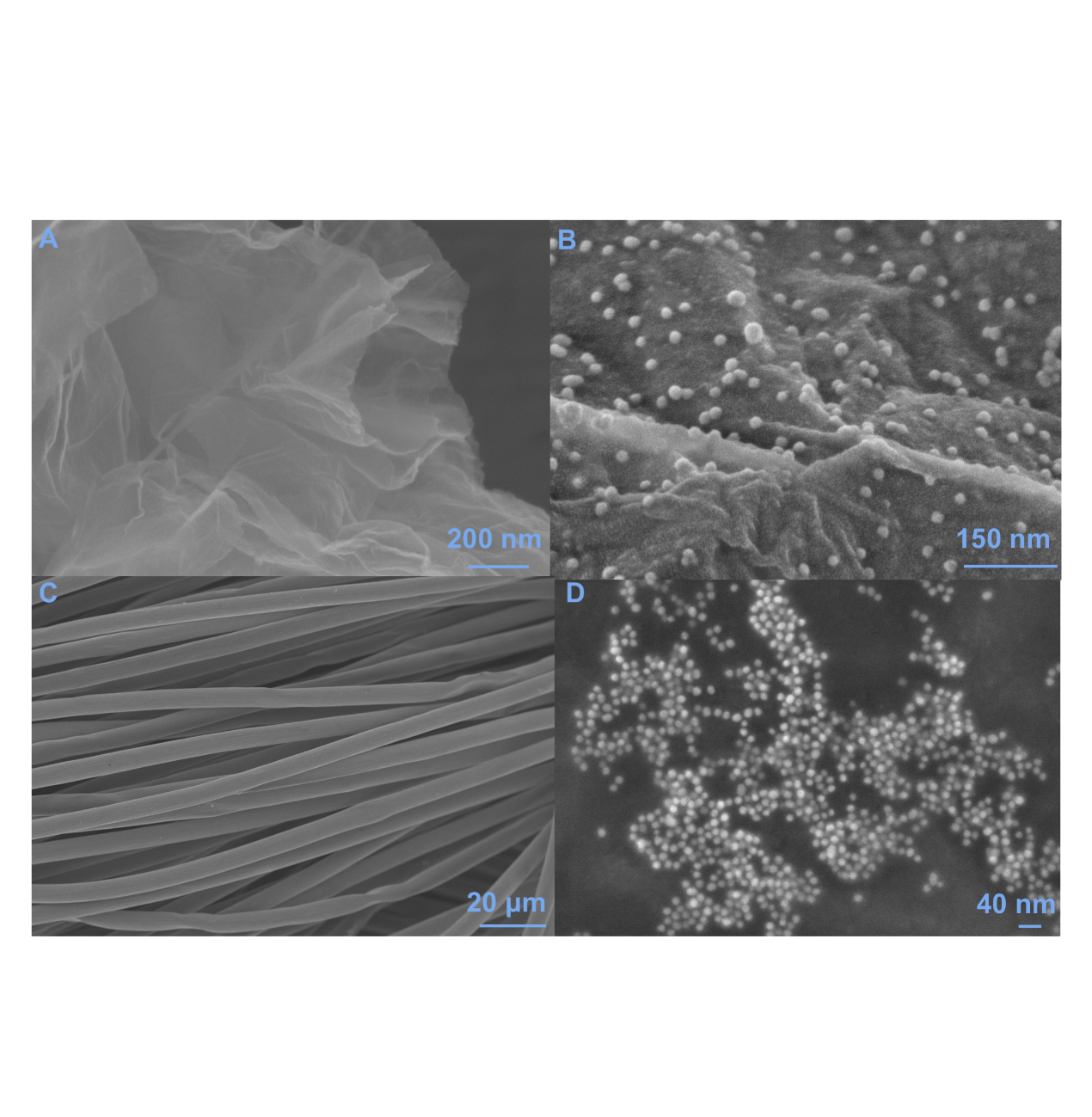
**

**Fig. S1.** (A, B) Scanning electron microscopy (SEM) images of GDY and GDY/Au NPs, (C, D) SEM images of CP and Au NPs


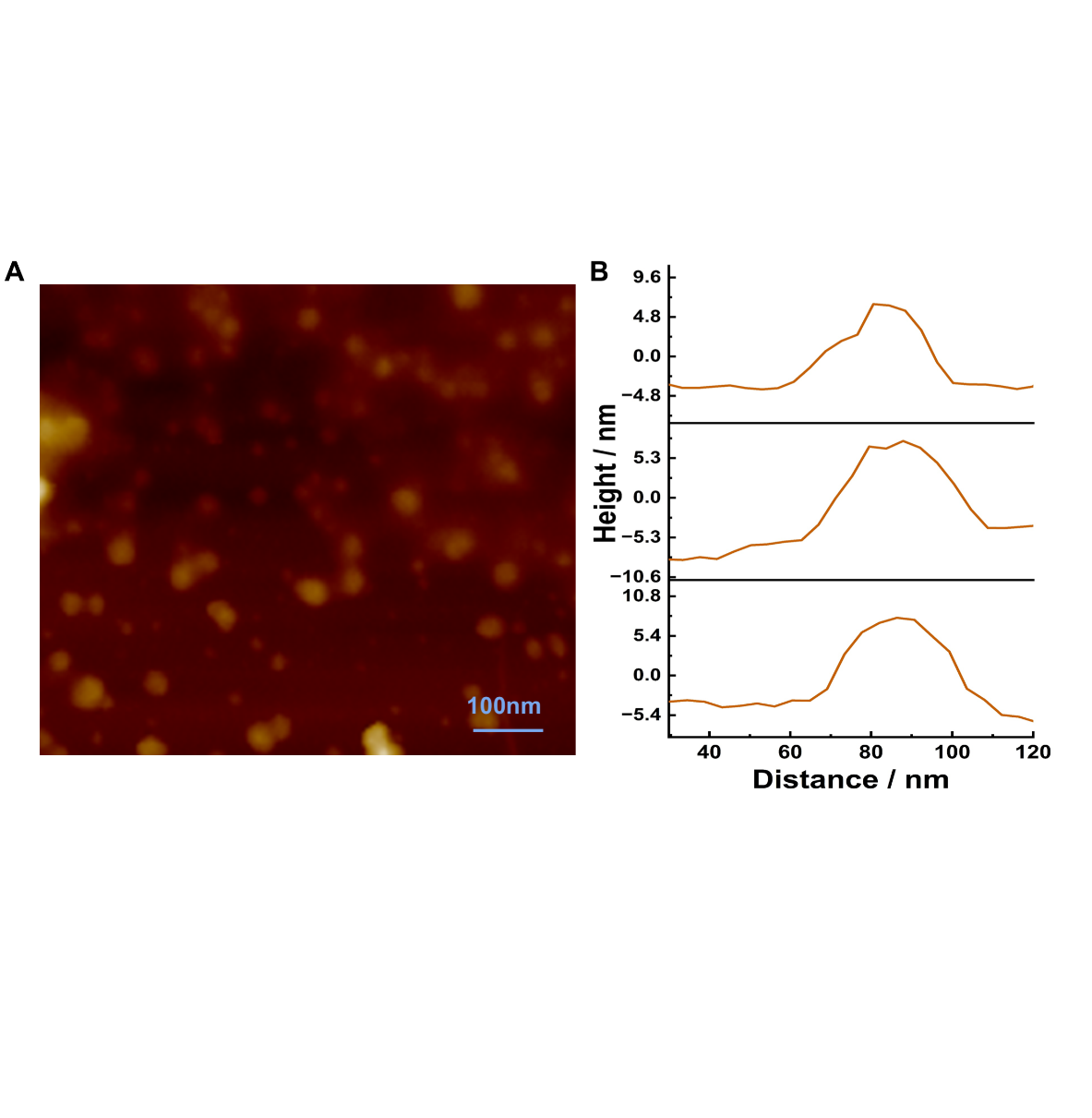


**Fig. S2.** AFM characterization of GDY/Au NPs.

**
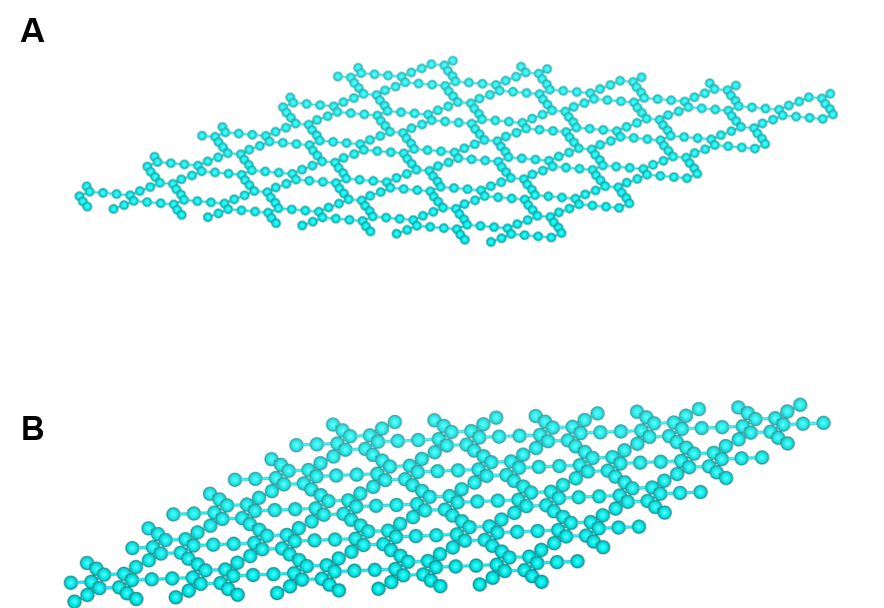
**

**Fig. S3.** Structural model diagram of GDY.


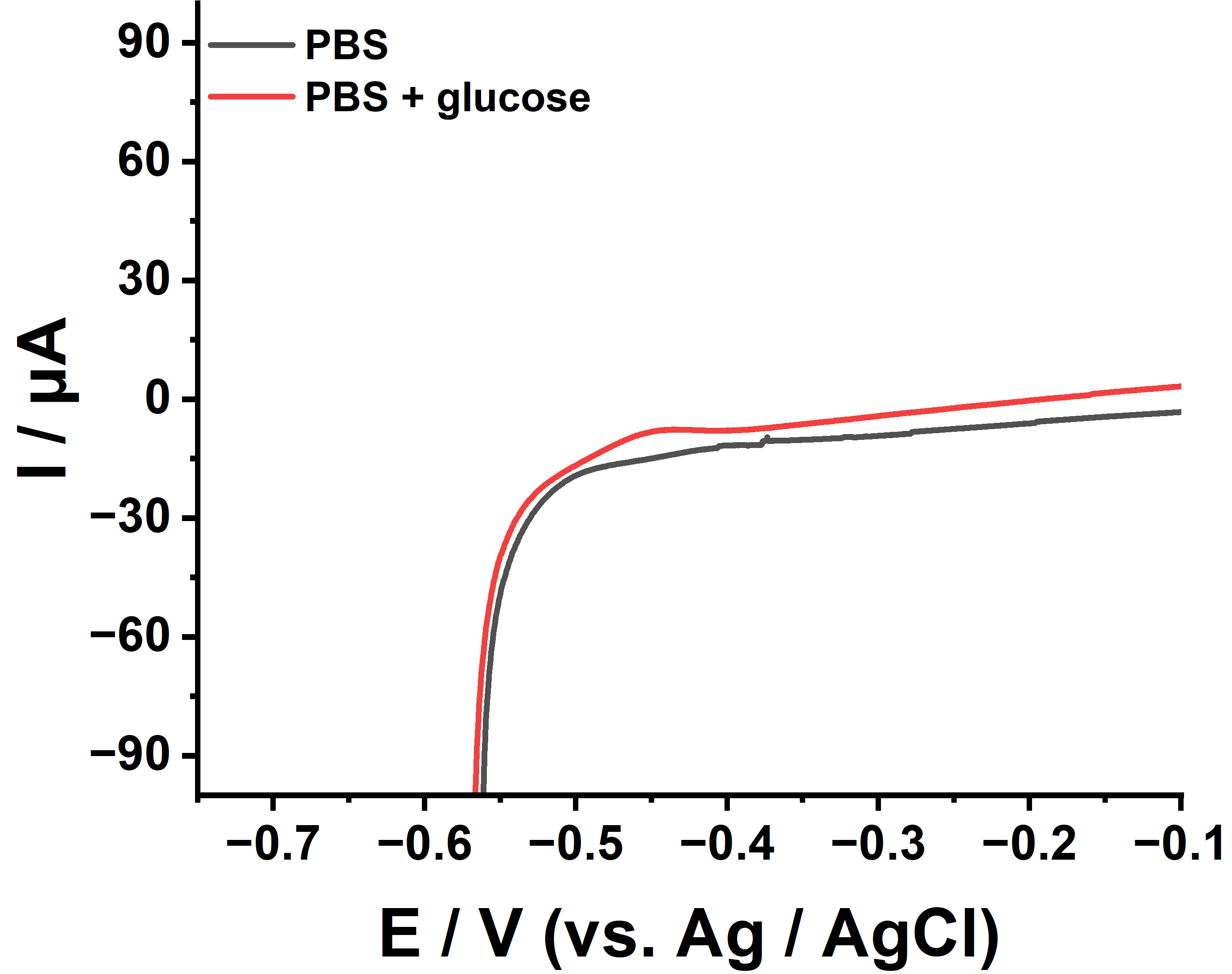


**Fig. S4.** LSV response of the bioanode in different electrolyte solutions.

**Fig. S5.** Open-circuit voltage (E^OCV^) of the biofuel cell measured without any external power supply
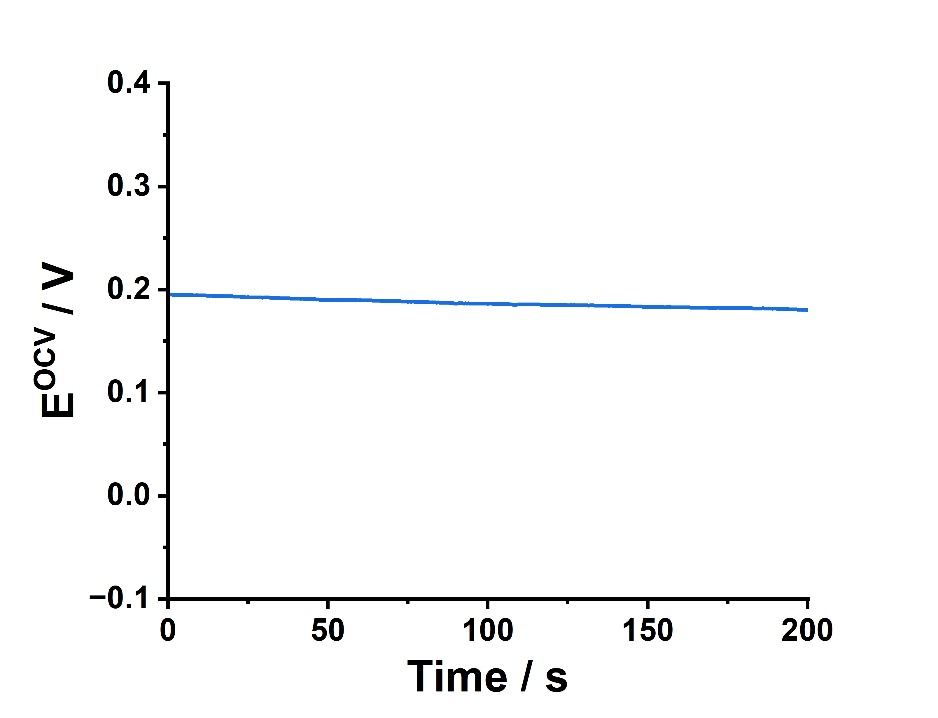


**Fig. S6.** Linear relationship between the signal intensity and the logarithm of the target concentration in the electrochemical mode.
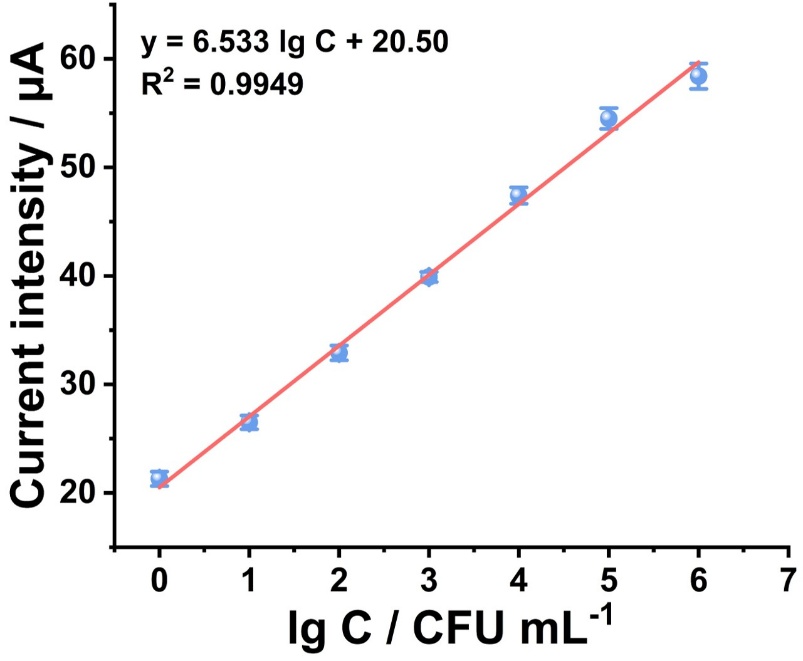


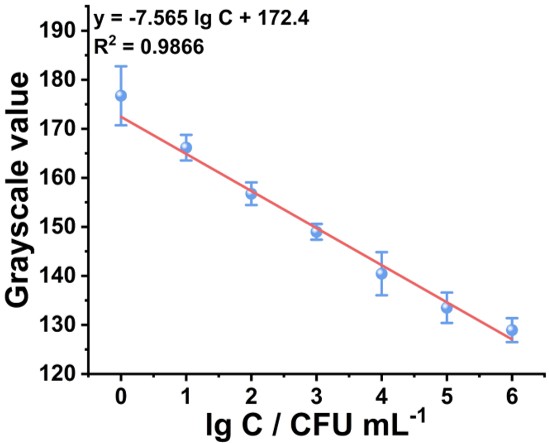


**Fig. S7.** Linear relationship between the signal intensity and the logarithm of the target concentration in the colorimetric mode.


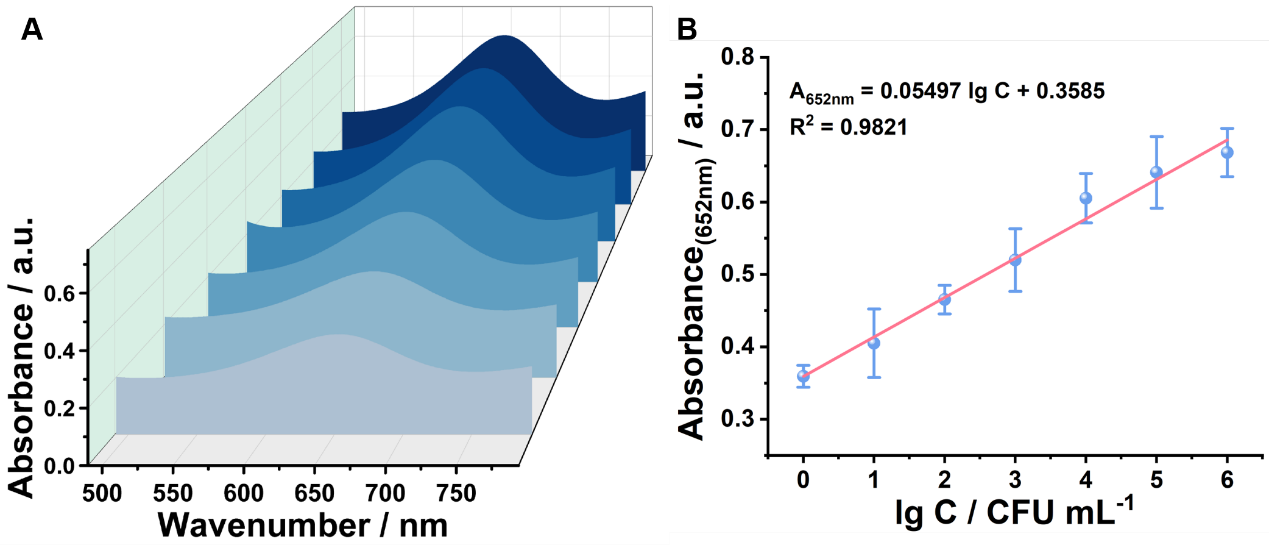


**Fig. S8.** (A) UV-vis absorbance of the self-powered biosensor in colorimetric mode at various concentrations of target bacteria, (B) Relationship between UV absorbance at 652 nm and the concentration of target bacteria, Study of sensor reproducibility in colorimetric mode based on UV-vis absorption spectra.

**Fig S9**. Temperature increase (ΔT) of PBS, TMB, oxTMB, and the sensing system with or without bacteria under NIR irradiation
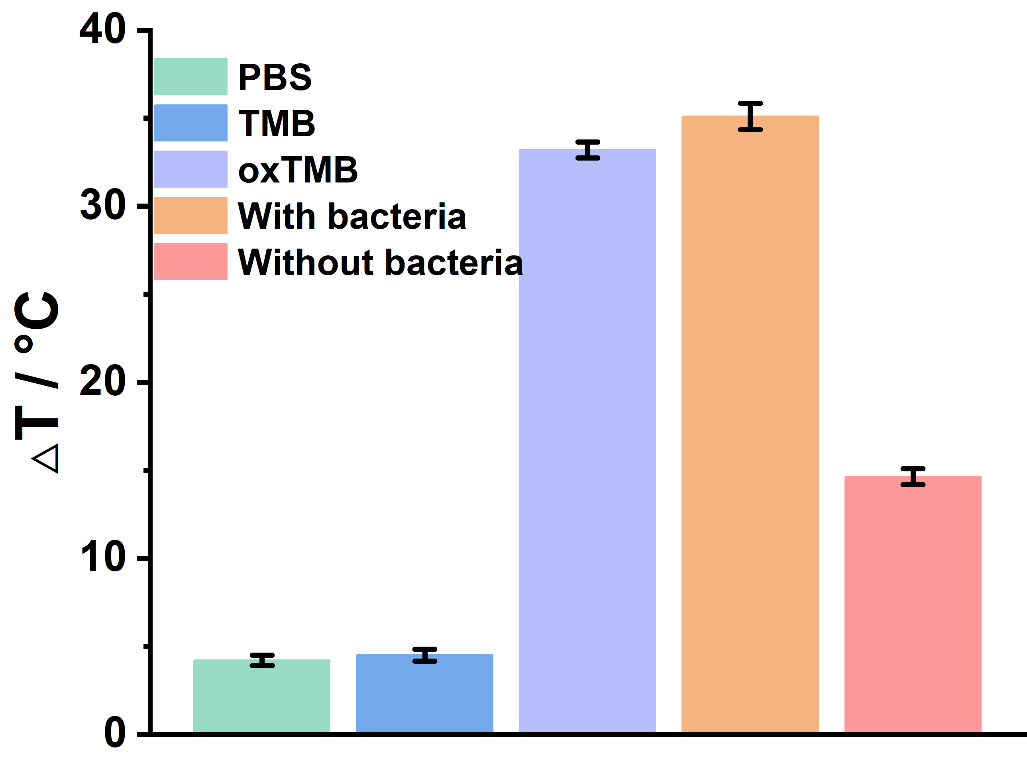
.

**
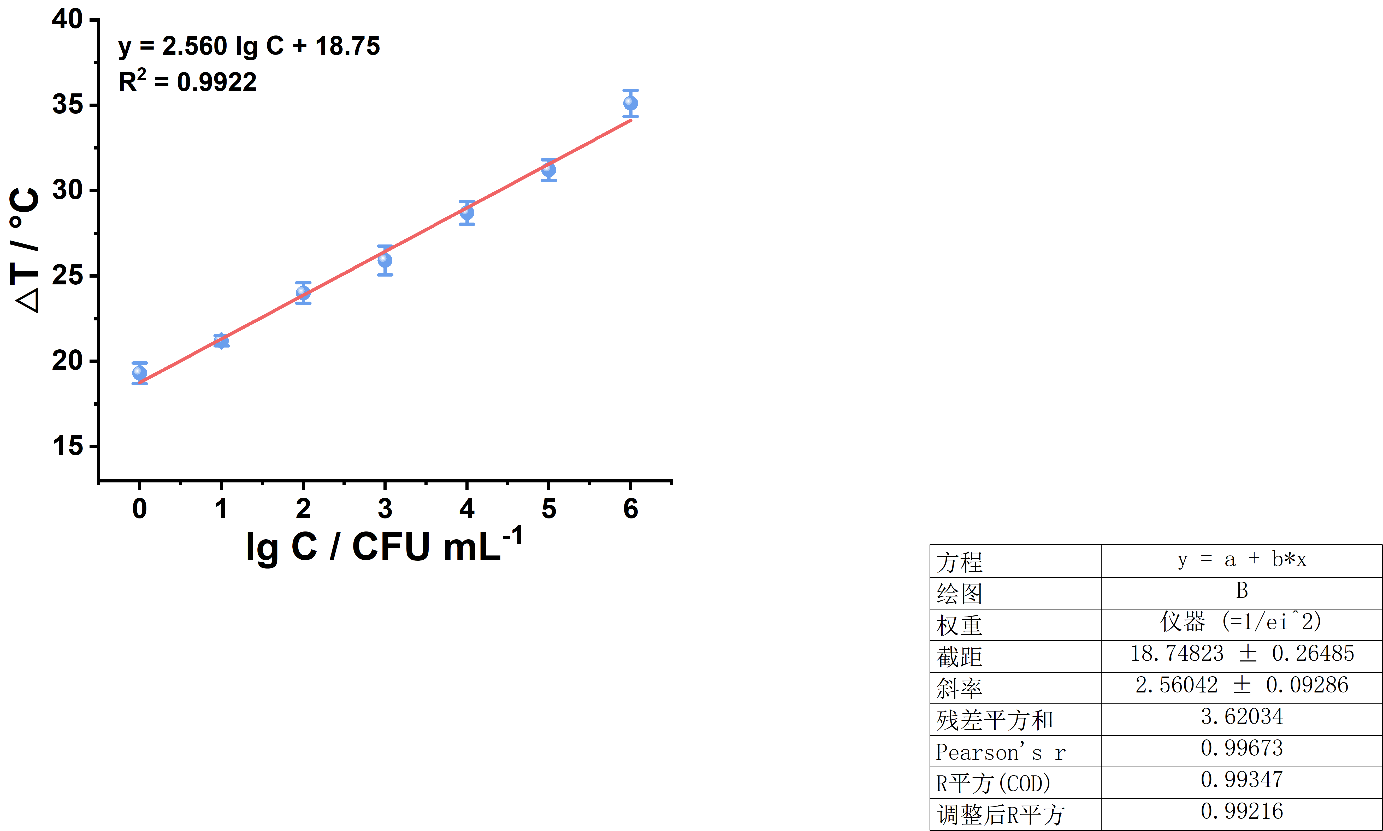
Fig. S10.** Linear relationship between the signal intensity and the logarithm of the target concentration in the thermal mode.

**
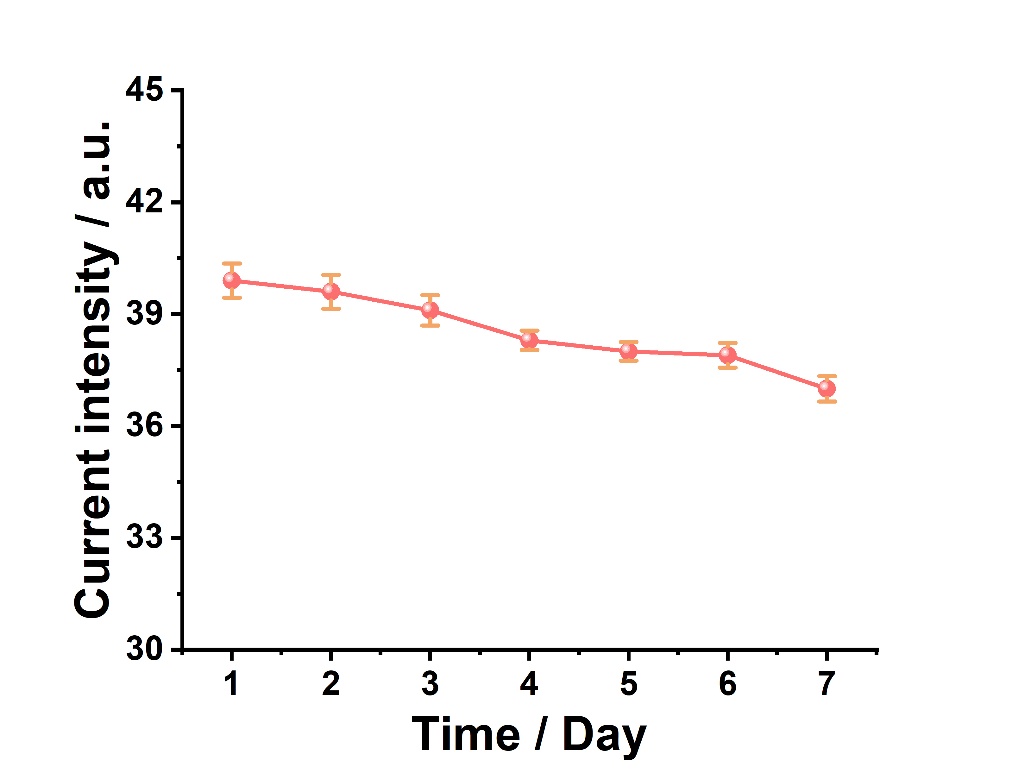
**

**Fig. S11**. Stability of the sensors stored at room temperature (25 °C)

**
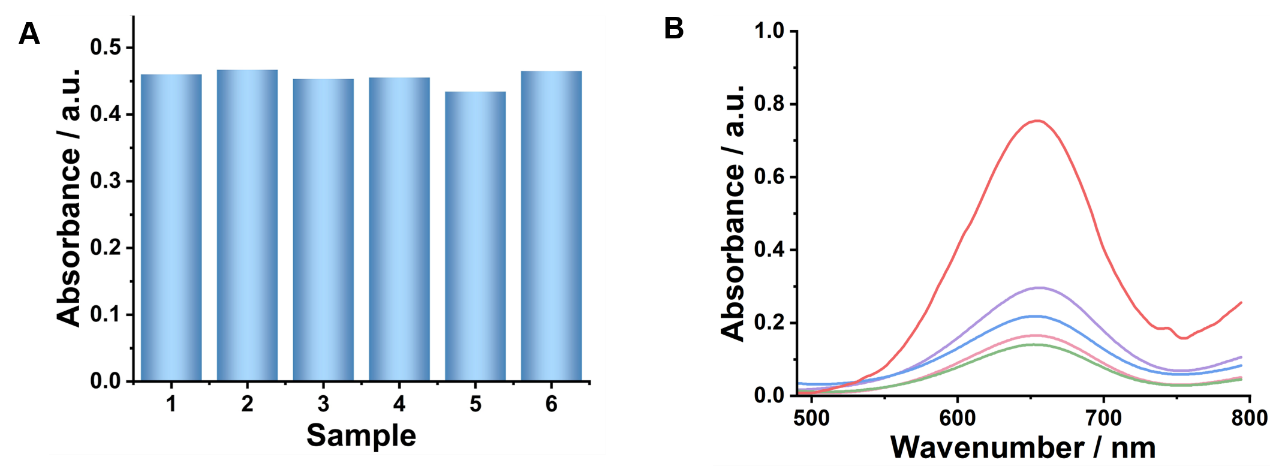
**

**Fig. S12.** (A) Vibrio parahaemolyticus concentration: 10^2^ CFU/mL), (B) UV-vis spectra of the reaction systems in the presence of different bacterial species.

**
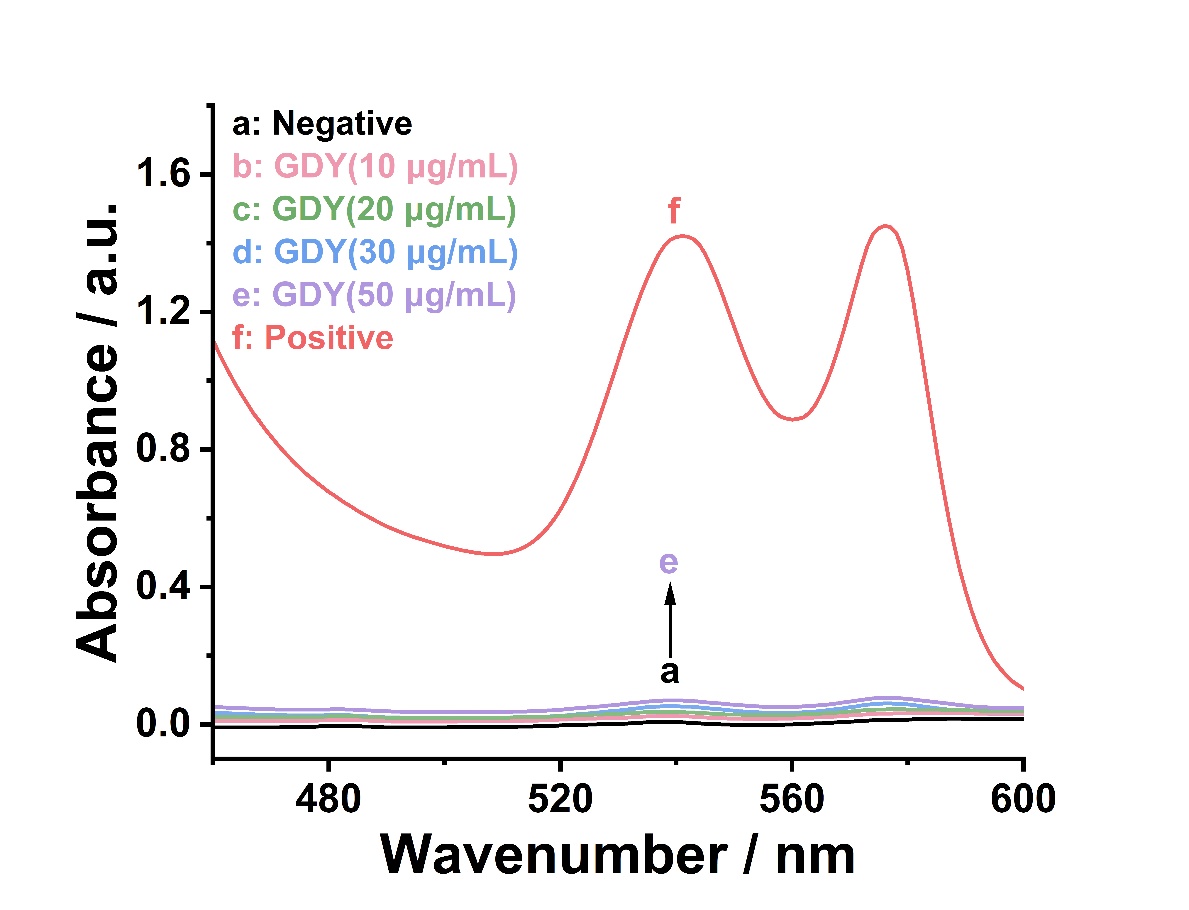
Fig. S13.** Hemolysis analysis based on UV-vis absorption spectra (analyzed using the absorbance at the β-band of oxyhemoglobin (HbO_2_) at 540 nm).

**Fig. S14**. UV-vis absorption spectra of red blood cell suspensions after incubation with GDY/AuNPs at different concentrations**
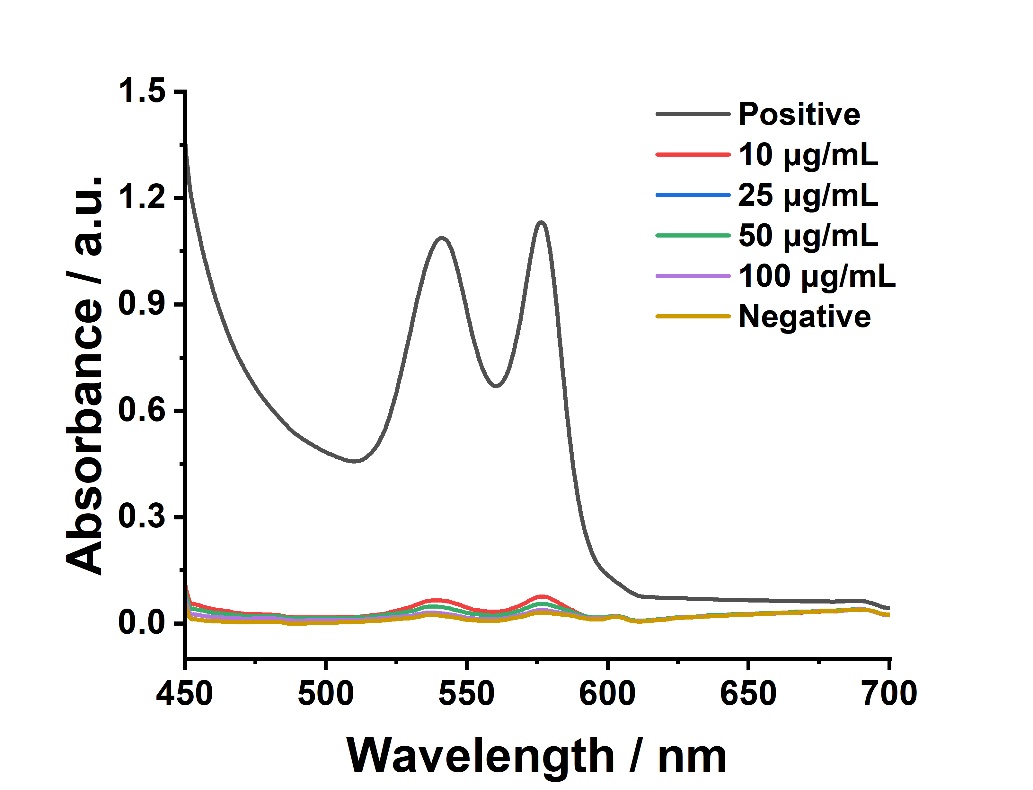
.**

**
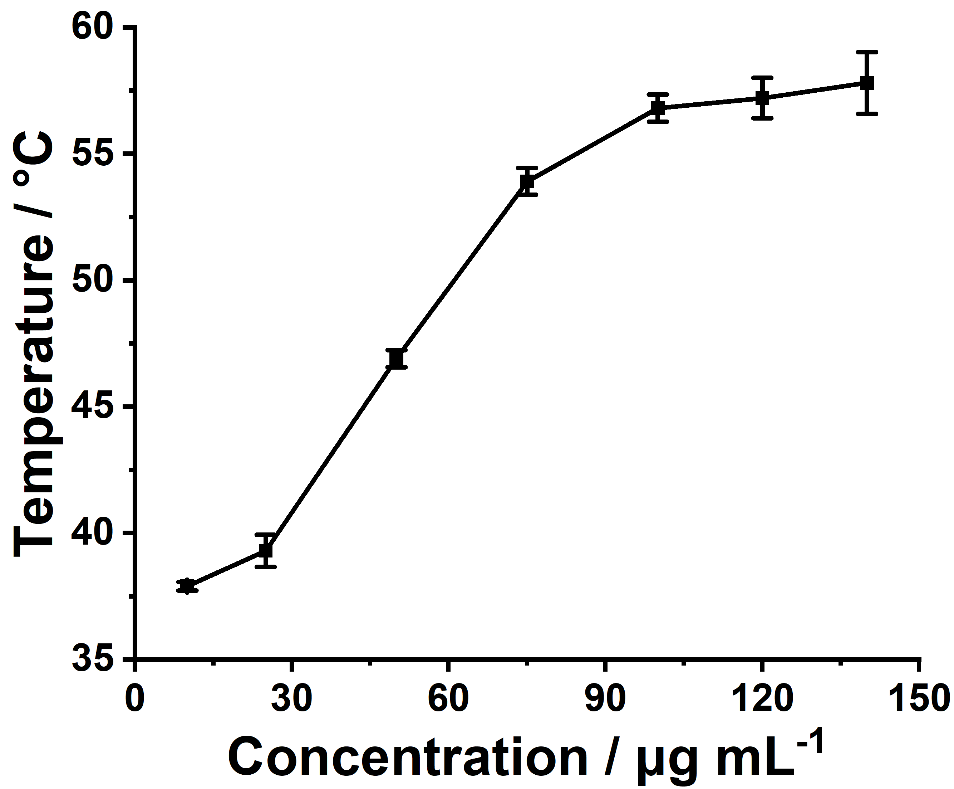
Fig. 15.** Optimization of GDY concentration

**
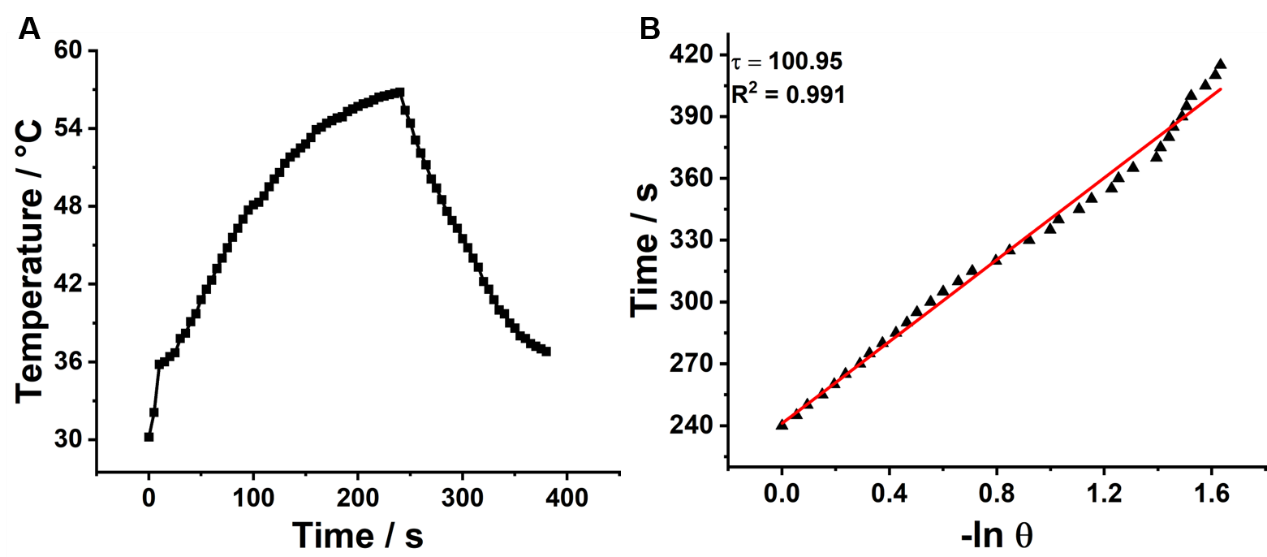
 Fig. S16.** (A, B) Photothermal performance of GDY/AuNPs under NIR irradiation.

**
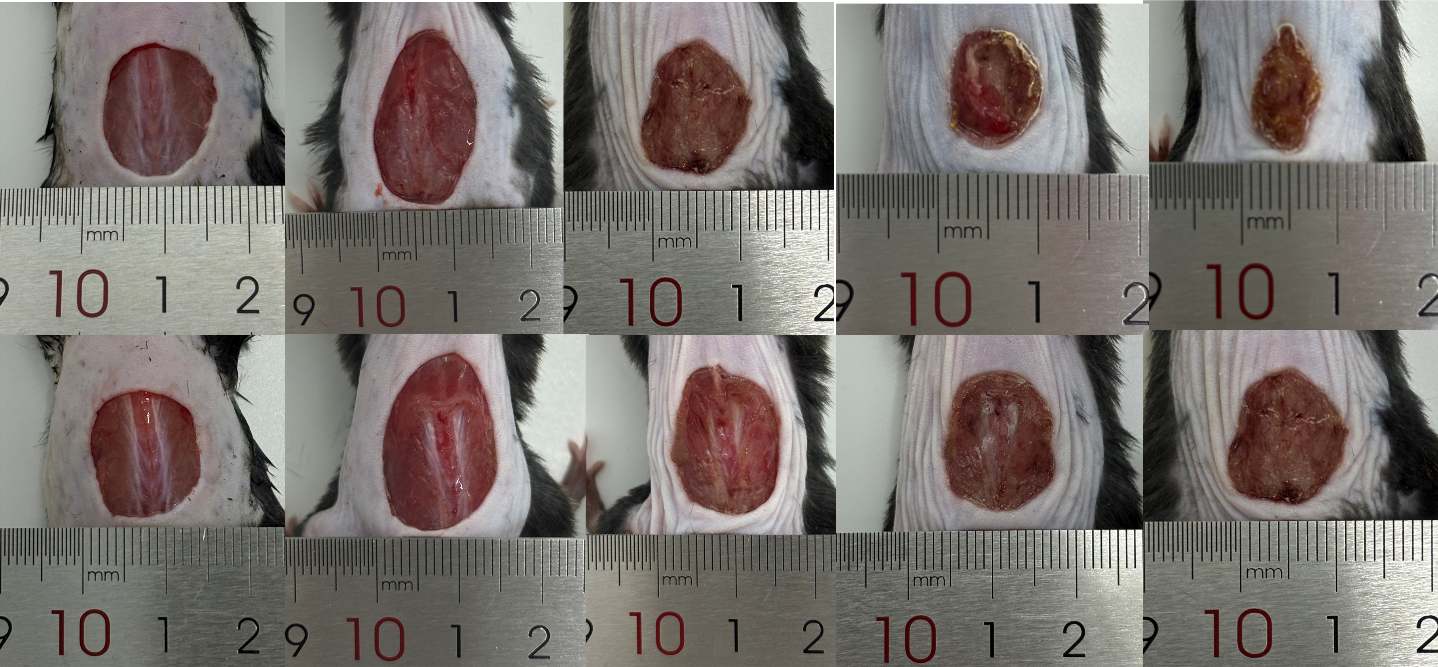
**

**Fig. S17.** Visualization of the wound healing process at different time points (Day 0, Day 1, Day 3, Day 5, Day 7) comparing the control group and the experimental group during treatment.

**
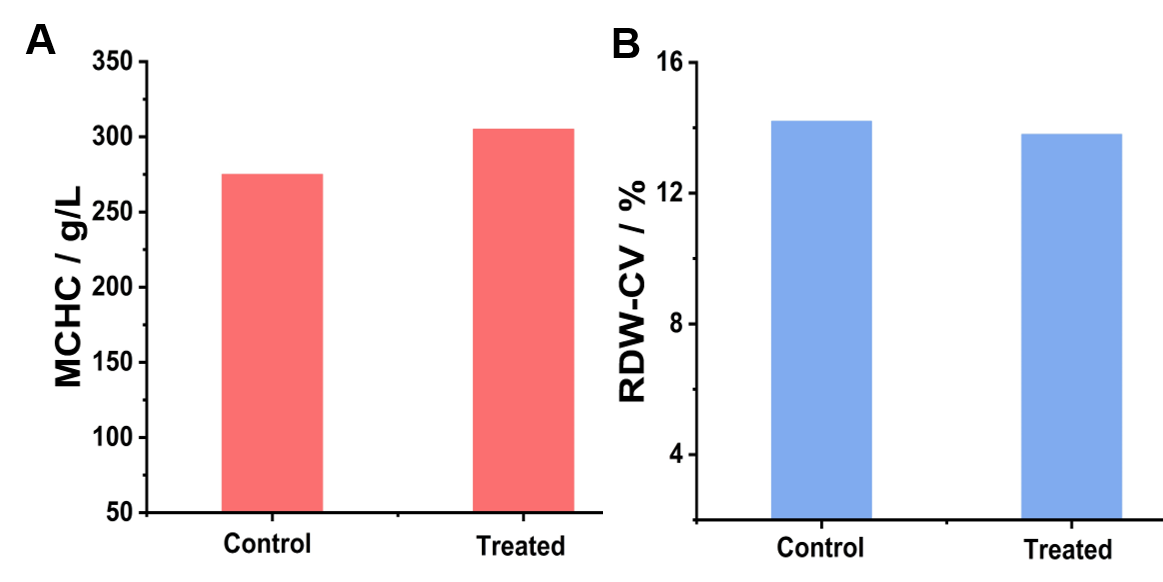
**

**Fig. S18.** (A, B) Red blood cell-related parameters, including (A) mean corpuscular hemoglobin concentration (MCHC) and (B) red blood cell distribution width-coefficient of variation (RDW-CV), were compared between the control and treatment groups.

**Fig. S19.**
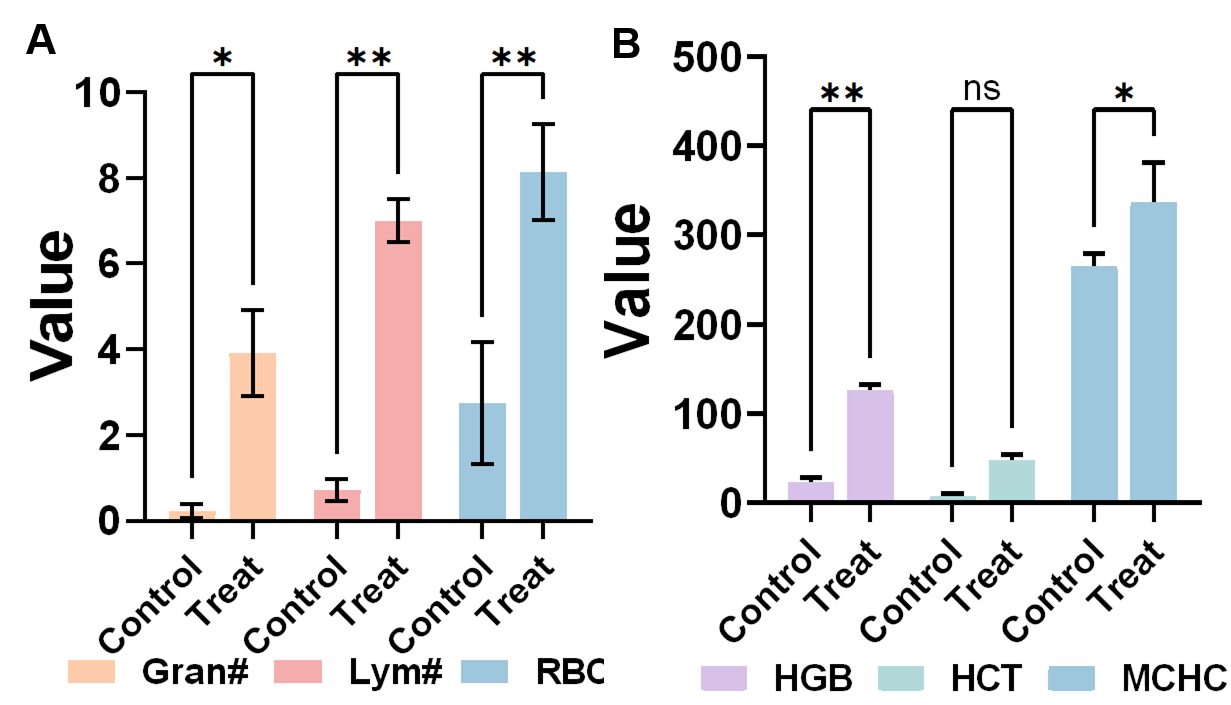
 Contrastive hematological parameter profiles of control and experimental cohorts.

**Table S1.** DNA sequences used in the experiments

| **Detection Methods** | **Materials** | **Targets** | **LODs**  **(CFU/mL)** | **Linear ranges** | **Detection**  **time** | **Ref.** |
| --- | --- | --- | --- | --- | --- | --- |
| Colorimetric-surface enhanced Raman sensor | Fe_3_O_4_@  MOF-GNs | VP | 7.00 / 9.00 | 10-10^5^ | 30 min | [3] |
| Photo-electrochemical  sensor | Bi_2_S_3_  GO/Cu_2_O | VP | 1.00 / 10.00 | 1-10^6^/ 10^2^-10^7^ | 2 h | [4] |
| Electrochemical aptasensor | MoS_2_ | VP | 5.74 | 10-10^6^ | 12 h | [5] |
| Self-powered electrochemical biosensor | GDY | VP | 0.34 / 0.41 / 0.78 | 1-10^6^ | 1 h | This  work |

| **Name** | **Sequences（from 5’ to 3’）** |
| --- | --- |
| HP | SH-(CH_2_)_6_-TTTAGACTGATCTACCTGAT-(CH_2_)_6_-NH_2_ |
| Apt-VP | ATAAGCATGAATTGACCAACCTAAACTTATTCATTTTCCAGCACCTCTAATATTACTGGC |
| crRNA | UAAUUUCUACUAAGUGUAGAUGCCAGUAAUAUUAGAGGUGC |

.

**Table S2.** Comparison of sensor performance with other reported sensors.

**Table S3.** Analytical results of V. parahaemolyticus detection in various water samples using the electrochemical method (n = 3).

| **Water specimen** | **V. parahaemolyticus concentrations (CFU/mL)** | | | **RSDs**  **(%)** | **Recoveries (%)** |
| --- | --- | --- | --- | --- | --- |
|  | Detected | Added | Found |  |  |
| 1 | Not detected | 5 | 4.77 | 3.26 | 95.40 |
|  |  | 10 | 9.66 | 4.43 | 96.60 |
| 2 | 5.5 | 5 | 9.92 | 1.67 | 94.48 |
|  |  | 10 | 15.83 | 4.42 | 102.1 |
| 3 | 6.5 | 20 | 26.88 | 5.89 | 101.8 |
|  |  | 50 | 56.94 | 3.36 | 100.8 |
| 4 | 19.5 | 50 | 68.76 | 4.89 | 98.94 |
|  |  | 100 | 120.70 | 2.88 | 101.0 |

**Table S4.** Analytical results for the detection of V. parahaemolyticus in different water samples using the colorimetric method (n = 3).

| **Water specimen** | **V. parahaemolyticus concentrations (CFU/mL)** | | | **RSDs**  **(%)** | **Recoveries (%)** |
| --- | --- | --- | --- | --- | --- |
|  | Detected | Added | Found |  |  |
| 1 | Not detected | 5 | 4.92 | 3.71 | 98.40 |
|  |  | 10 | 10.05 | 6.29 | 100.5 |
| 2 | 6.0 | 5 | 11.80 | 5.55 | 107.3 |
|  |  | 10 | 16.31 | 4.06 | 101.9 |
| 3 | 6.8 | 20 | 26.14 | 4.82 | 97.54 |
|  |  | 50 | 57.35 | 2.15 | 101.0 |
| 4 | 20.0 | 50 | 72.00 | 4.86 | 102.9 |
|  |  | 100 | 122.71 | 6.92 | 102.3 |

**Table S5.** Analytical results for the detection of V. parahaemolyticus in different water samples using the specific heat method (n = 3).

| **Water specimen** | **V. parahaemolyticus concentrations (CFU/mL)** | | | **RSDs**  **(%)** | **Recoveries (%)** |
| --- | --- | --- | --- | --- | --- |
|  | Detected | Added | Found |  |  |
| 1 | Not detected | 5 | 5.13 | 4.13 | 102.6 |
|  |  | 10 | 10.22 | 4.29 | 102.2 |
| 2 | 6.0 | 5 | 11.18 | 4.30 | 101.6 |
|  |  | 10 | 16.02 | 4.33 | 100.1 |
| 3 | 6.5 | 20 | 25.89 | 4.17 | 97.70 |
|  |  | 50 | 56.45 | 4.31 | 99.91 |
| 4 | 20.0 | 50 | 71.88 | 7.33 | 102.3 |
|  |  | 100 | 121.18 | 2.10 | 101.0 |

**Table S6**. Spike recovery results for the detection of *Vibrio parahaemolyticus* in diluted human serum samples (n = 3).

| **Water specimen** | **V. parahaemolyticus concentrations (CFU/mL)** | | **RSDs**  **(%)** | **Recovery**  **(%)** |
| --- | --- | --- | --- | --- |
|  | Added | Found |  |  |
| 1 | 5 | 5.62 | 5.27 | 112.4% |
|  | 10 | 10.75 | 3.83 | 107.5% |
| 2 | 10 | 10.07 | 4.26 | 100.7% |
|  | 20 | 19.84 | 1.65 | 99.20% |
| 3 | 20 | 20.52 | 3.74 | 102.6% |
|  | 50 | 50.91 | 4.72 | 101.8% |
| 4 | 50 | 48.33 | 5.11 | 96.66% |
|  | 100 | 102.51 | 4.78 | 102.5% |

**References**

1. J. Xu, Y. J. Li, F. T. Wang, H. F. Yang, K. J. Huang, R. Cai,; W. H. Tan, A Smartphone-Mediated “All-In-One” Biosensing Chip for Visual and Value-Assisted Detection. *Anal. Chem.* **96**, 15780-15788 (2024).

2. J. Xu, Y. B. Liu, X. Q. Luo, Y. J. Li, Y. Q. Xing, K. J. Huang, Visual self-powered platform for ultrasensitive heavy metal detection designed on graphdiyne/graphene heterojunction and DNAzyme-triggered DNA circuit strategy. *Chem. Eng. J.* **485**, 150151 (2024).

3. J. Li, X. F. Lin, J. J. Wu, D. C. Ying, N. Duan, Z. P. Wang, S. J. Wu, Multifunctional magnetic composite nanomaterial for Colorimetric-SERS Dual-Mode detection and photothermal sterilization of Vibrio parahaemolyticus. *Chem. Eng. J.* **477**, 147113 (2024).

4. Q. Q. Zhang, T. T. Zhai, Y. X. Guo, Y. F. Weng, N. Zhou, H. Lin, H. Tan, K. N. Lu, Y. Y. Zhou, Faraday cage-type photocurrent polarity switching photoelectrochemical sensing platform for highly selective and sensitive detection of Vibrio parahaemolyticus. *Food Chem.* **475**, 143275 (2025).

5. H. Jiang, Z. K.Sun, Q.Guo, X.Weng, Microfluidic thread-based electrochemical aptasensor for rapid detection of Vibrio parahaemolyticus. *Biosens. Bioelectron.* **182**, 113191 (2021).
